# Functional network analysis in hepatolithiasis: identifying novel therapeutic targets through whole-exome sequencing

**DOI:** 10.7717/peerj.21059

**Published:** 2026-04-09

**Authors:** Dan Tang, Xuanyu Gu, Dan Liu, Jiali Yang, Lijin Zhao

**Affiliations:** 1 Suzhou Medical College of Soochow University, Suzhou, People’s Republic of China, Suzhou, China; 2 Department of General Surgery, Digestive Disease Hospital, Affiliated Hospital of Zunyi Medical University, Zunyi, Guizhou, China; 3 Hepatobiliary and Pancreatic Surgery, the Second Affiliated Hospital of Zunyi Medical University, Zunyi, Guizhou, China

**Keywords:** Hepatolithiasis, Hepatatrophia, Whole-exome sequencing, Mutation analysis

## Abstract

**Background:**

Hepatolithiasis (HL) is a prevalent condition in hepatobiliary surgery, often complicated by hepatatrophia. This study aimed to identify gene mutations in HL specimens with hepatatrophia and construct a mutation landscape using whole-exome sequencing (WES).

**Methods:**

Genetic variants and copy number variations (CNVs) were compared between HL (*n* = 10) and normal control (NC, *n* = 10) specimens, encompassing 20 participants in total, using WES data. Hub genes within HL specimens were identified *via* the GeneCards database. The mutation frequencies and interaction patterns of these hub genes were explored, and their potential molecular mechanisms were assessed. A competing endogenous RNA (ceRNA) network and a transcription factor (TF)-mRNA network were established to clarify the regulatory mechanisms of hub genes. Therapeutic drugs targeting these hub genes were predicted using the DrugBank database, and molecular docking assessed the binding energies. Finally, hub gene expression was validated through reverse-transcription quantitative PCR (RT–qPCR).

**Results:**

Seven hub genes (*TMEM150B*, *TNIP1*, *ATRN*, *FAAH*, *FBXW4*, *RAX*, and *WNT8B*) were identified as uniquely mutated in HL specimens, with *TMEM150B* exhibiting the highest mutation frequency. The concurrent mutations of these genes are likely associated with the pathogenesis of HL combined with hepatatrophia. Functional enrichment analysis indicated that these hub genes are involved in the inflammatory response and Wnt signaling pathway. The ceRNA-TF network revealed that 296 lncRNAs (*e.g*., C1RL-AS1) regulate six miRNAs (*e.g*., hsa-miR-195-5p) targeting four hub genes, while 19 TFs (*e.g*., NFYA, HINFP) also regulate these genes. Furthermore, fostamatinib exhibited the strongest binding affinity with *FAAH*, with a binding energy of −9.2 kcal/mol, suggesting that it is a promising candidate for further investigation. RT–qPCR confirmed that *TMEM150B* and *FAAH* expression levels were reduced in HL samples, while *TNIP1*, *ATRN*, *FBXW4*, and *WNT8B* were upregulated. Expression of *RAX* was not reliably detectable.

**Conclusion:**

This study identified *TMEM150B*, *TNIP1*, *ATRN*, *FAAH*, *FBXW4* and *WNT8B* as key genes in the development of HL through inflammatory response and Wnt signaling pathways, providing new theoretical insights into therapeutic mechanisms.

## Introduction

Hepatolithiasis (HL) is a condition characterized by the formation of stones within the liver, typically presenting as brown pigment stones (calcium bilirubinate stones) with a higher cholesterol content. It is prevalent in Southeast Asia and East Asia but rare in Western countries (Lorio et al., 2020; Shoda, Tanaka & Osuga, 2003). Hepatic atrophy is a common complication of HL (Feng, Xia & Yan, 2016). Obstruction caused by HL, coupled with local inflammation triggered by the stones, leads to hyperplasia and fibrosis of the bile ducts downstream of the obstruction. Impaired bile flow results in atrophy and fibrosis in certain liver regions, while other segments experience compensatory growth, causing dysregulation of liver size and shape. In severe cases, this can lead to malignant transformation of the atrophic tissue (Li et al., 2009a). Currently, hepatectomy is considered the most effective treatment for HL, but the underlying etiology remains poorly understood, complicating the management of hepatobiliary atrophy with cholangiolithiasis (Leung & Yu, 1997).

Whole-exome sequencing (WES) is a genomic technique that employs target sequence capture technology to capture all exonic DNA regions and conduct high-throughput sequencing. It provides high sensitivity in identifying low-frequency and rare mutations associated with diseases (Ng et al., 2010; Veltman & Brunner, 2012). Previous studies have identified specific genes linked to HL pathogenesis, such as mutations in the *ABCB11* gene, which affect the expression of its encoded protein in patients with primary HL (de Sena Brandine & Smith, 2019). However, genetic mutation analysis in hepatobiliary atrophy with cholangiolithiasis remains limited.

In this study, peripheral blood samples from healthy individuals and patients with hepatobiliary atrophy and cholangiolithiasis were collected to perform WES. The mutational spectrum was mapped to analyze the genetic variant landscape and identify significant mutant genes in these patients, providing a theoretical foundation for a deeper understanding of the pathogenetic mechanisms and potential therapeutic targets for the disease.

## Materials and Methods

### Whole exome sequencing

Peripheral blood samples were collected from 10 patients with HL complicated by liver atrophy and 10 normal control (NC) individuals. The samples were sent to Sangon Biotech (Shanghai, China) for WES. Informed consent was obtained from all participants, and ethical approval was granted by the Ethics Committee of Zunyi Medical University (approval number: <2019>1-058). Specimens with high-quality reads were processed for further analysis using fastq (v0.11.9) (Adhikari et al., 2022) and trimmomatic (v0.39) (Jindal et al., 2022) software based on the original WES FASTQ data. Quality-controlled FASTQ files were aligned to the reference genome (hg19) using BWA software (0.7.17) (Shoda et al., 1993), and the results were ranked with SAMtools (Xiao et al., 2021). It should be noted that the WES data in this study were derived from peripheral blood samples; therefore, the identified variants include germline and potentially hematopoietic system-derived genetic variants, which may not directly represent liver-specific mutational patterns.

### Genetic variant analysis

To obtain genetic variant data, several processing steps were applied to the WES data. Initially, the GATK package (v3.8.0) (Shao et al., 2019) was used to mark duplicate sequences and detect mutations in single nucleotide polymorphisms (SNPs) and insertions/deletions (indels). The variation data were then filtered using the VQSR method, based on known human variants from the Single-Nucleotide Polymorphism database (dbSNP, https://www.ncbi.nlm.nih.gov/snp/). The annotated variation data were processed with annovar software and converted into a maf file. Mutation landscape analysis, including mutation status and types, was performed using maftools (v2.10.0) (Mrschtik & Ryan, 2016). The frequency of different mutation types (missense, nonsense, stop-loss, frameshift mutations, indels, splice sites, TMB, *etc*.) was quantified using maftools software (v2.10.0) (Mrschtik & Ryan, 2016).

### Analysis of genetic variant patterns and copy number variation

To investigate genetic variant patterns, mutation data were analyzed based on the frequency of single-base substitutions at the 5′- and 3′-end bases. Co-mutation analysis was performed to identify enrichment patterns of mutations in each sample by comparing them with mutational signatures v3 in the COSMIC database. Cosine similarity was calculated to identify features with high similarity to those in the study, which were visualized in a heatmap. Furthermore, the GISTIC2 algorithm (v2.0) (Mirinezhad et al., 2021) (threshold = 0.99) was used to detect frequently altered genomic regions in HL and NC specimens.

### Analysis of hub gene mutation

Genes mutated in more than 5 HL specimens were selected as hub genes for further analysis (excluding synonymous mutations). Information on these hub genes was retrieved from the GeneCards database (http://www.genecards.org). Co-occurrence and mutual exclusivity of these hub genes were further explored.

### Functional annotations of hub genes

Gene Ontology (GO) and Kyoto Encyclopedia of Genes and Genomes (KEGG) enrichment analyses were performed using the Metascape network (https://metascape.org). Cytoscape software (v 3.8.2) was employed to visualize the regulatory network of hub genes and the enriched pathways.

### Protein-protein interaction and regulatory networks of hub genes

The protein-protein interaction (PPI) network was constructed using the Gene MANIA database (http://genemania.org). MicroRNAs (miRNAs) targeting the hub genes were predicted by overlapping miRNAs obtained from the miRWalk database (http://mirwalk.uni-hd.de/) and the Starbase database (http://starbase.sysu.edu.cn/). Long non-coding RNAs (lncRNAs) targeting these miRNAs were identified by intersecting lncRNAs retrieved from Starbase and MirNet software (https://www.mirnet.ca). Transcription factors (TFs) targeting the hub genes were predicted using NetworkAnalyst (http://www.networkanalyst.ca/faces/home.xhtml), with intensity signals <500 and scores <1. All networks were constructed using Cytoscape software (v3.8.2) (Oh et al., 2016).

### Drug prediction of hub genes and molecular docking

Potential therapeutic drugs targeting the hub genes were identified *via* the DrugBank database (www.drugbank.ca). Drug structures were predicted using the PubChem database (https://pubchem.ncbi.nlm.nih.gov). The protein structures of the hub genes were extracted from the UniProt database (https://www.uniprot.org) based on drug structure information. Finally, molecular docking was performed using the DockThor website (v1.5.7) (Restuadi et al., 2022).

### Molecular dynamics simulations

To further investigate how fostamatinib influences the molecular mechanism of HL through regulation of hub genes, the conformation with the highest (most stable) binding energies to *FAAH* was selected for molecular dynamics (MD) simulations. GROMACS (version 2024.2) software was used to conduct a 100 ns MD simulation (Páll et al., 2020), providing additional validation for the docking results. Prior to the simulation, pre-equilibration was performed in two stages to optimize the orientation of the molecules and minimize errors in the dynamics simulation. In the first stage, the NVT system was used to simulate and equilibrate at 300 K for 100 ps to stabilize the system temperature. In the second stage, the NPT system was employed to simulate and equilibrate at 1 bar for 100 ps to stabilize system pressure. The “AMBER14SB” force field and the AMBER gaff force field were applied to generate the parameters and topology files for proteins and small molecule ligands, respectively. To mitigate the long-range interactions introduced by periodic boundary conditions, these conditions were optimized and filled with water molecules. To ensure the system was electrically neutral, some solvent water molecules were replaced by Na^+^ and Cl^−^ ions at a concentration of 0.15 mol/L. Energy minimization of the entire system was performed using the steepest descent method. The Root Mean Square Deviation (RMSD), Root Mean Square Fluctuation (RMSF), and radius of gyration (Rg) were calculated to examine positional changes between the conformations during the simulation and the initial conformation, protein amino acid flexibility, and the compactness of the protein structure throughout the simulation. In the protein-small molecule ligand interaction simulation, variations in hydrogen bond numbers reflected the stability and dynamics of their interactions. Changes in bond energy served as a critical indicator for assessing binding strength, displaying energy fluctuations during protein-ligand binding, thereby providing insights into their interaction mechanisms. The interfacial area, a key physical parameter, was used to evaluate the extent and mode of interaction between the protein and small molecule ligand. Additionally, the distance between the binding site of the small molecule and the protein’s amino acid residues was monitored as a dynamic parameter, reflecting binding stability, interaction mechanisms, and conformational changes.

### Reverse-transcription quantitative PCR

To further investigate the role of hub genes in HL, the expression of hub genes was validated using reverse-transcription quantitative PCR (RT-qPCR). A total of 20 clinical blood samples were obtained for this experiment from normal individuals and HL patients at the Department of Hepatobiliary Surgery, Affiliated Hospital of Zunyi Medical University, and the Department of Hepatobiliary Surgery, Second Affiliated Hospital of Zunyi Medical University. The samples consisted of 10 normal and 10 HL specimens, with 3 mL of blood collected for each sample. This study was performed in accordance with the Declaration of Helsinki and approved by the Ethics Committee of Zunyi Medical University (Approval No. <2019>1-058). Written informed consent was obtained from all included patients prior to study participation.

Total RNA was extracted from peripheral blood mononuclear cells (PBMCs) using TRIzol reagent (Accurate Biological, China) following the manufacturer’s protocol. PBMCs were isolated from whole blood by density gradient centrifugation at 2,000 g for 20 min at room temperature. After PBMC collection and washing, total RNA was extracted with TRIzol-chloroform phase separation, followed by isopropanol precipitation. The RNA pellet was washed twice with 75% ethanol, air-dried, and resuspended in RNase-free water. RNA concentration and purity were assessed using a NanoPhotometer N50 (Thermo Fisher, Waltham, MA, USA), and samples with an A260/A280 ratio between 1.8 and 2.0 were used for subsequent analysis. cDNA was synthesized from 2 µg total RNA using the SureScript First-strand cDNA Synthesis Kit (Servicebio, Hubei, China) in a 20 µL reaction volume according to manufacturer instructions (Tables S1, S2). RT-qPCR was performed with the Aptamer PCR SYBR Green Master Mix (Novogene, Beijing, China) using *GAPDH* as the endogenous control. Primer sequences are listed in Table 1. Each 10 µL reaction contained 3 µL diluted cDNA, 5 µL of 2× Universal Blue SYBR Green qPCR Master Mix (Thermo Scientific, Waltham, MA, USA), and 1 µL each of forward and reverse primer (from 10 µM stock solutions), yielding a final concentration of 1 µM for each primer. Reactions were run in triplicate on a BIO-RAD CFX Connect Real-Time PCR System (v 3.1) using the program in Table S3. No-template controls showed no amplification. Data were analyzed using the 2^−ΔΔCT^ method, and statistical analysis was performed with GraphPad Prism 5 software. Instrument details used in the experiment are provided in Table S4.

**Table 1:** PCR primer sequence of hub genes. Gene: Names of hub genes, including *TMEM150B*, *TNIP1*, *ATRN*, *FBXW4*, *FAAH*, *WNT8B*, and *GAPDH* as the reference gene. Forward Primer: Forward primer sequences, which are used to complementarily bind to specific regions of the template DNA in PCR reactions and initiate DNA synthesis. Reverse Primer: Reverse primer sequences, which work in conjunction with forward primers to define the region of the DNA fragment to be amplified in PCR reactions.

Gene	Forrward primer	Reverse primer
*TMEM150B*	CTCCAGACACCCAACCTACC	GCTCTCTCCAAGCTTCCTGG
*TNIP1*	CATTGTGTGCCAGTAAGTGCC	CCTCCAGAAGACCCCTCCAA
*ATRN*	ACGACATGCCCAGAAGTGAC	GCCTGCTGAGTGAAAGCAAG
*FBXW4*	GTCATGTATGAGTCCCCTTTCAC	ACAACACCGTAGTAGGAGGAAC
*FAAH*	GAAGTCTCGTTCGGCTGGAA	TCCCCAAAGTAGCCCCTGTA
*WNT8B*	AGAAGTACCACGCAGCACTC	GCGTTTTGTTCTCCAGGCAG
*GAPDH*	CGAAGGTGGAGTCAACGGATTT	ATGGGTGGAATCATATTGGAAC

### Statistical analysis

Statistical analyses and data visualization were primarily conducted using R software (v 4.1.1). Genetic variant and CNV analyses were performed with the GATK package (v 3.8.0), maftools (v 2.10.0), and GISTIC2 (v 2.0). Functional enrichment and network analyses were performed *via* Metascape, GeneMANIA, and Cytoscape (v 3.8.2). For RT–qPCR data, relative gene expression was calculated using the 2^−ΔΔCT^ method, and statistical significance between groups was evaluated using a two-tailed Student’s t-test in GraphPad Prism 5. A *p*-value < 0.05 was considered statistically significant.

## Results

### WES data processing

The analysis of FASTQ data from the 20 specimens revealed high read quality for subsequent analyses (Table S5). After quality control (QC), the comparison results indicated that the mapped reads for all specimens exceeded 97%, suggesting that the majority of reads aligned with the reference genome without interference from exogenous RNAs (Table S6). Furthermore, the coverage of each specimen at ≥10× was greater than 96%, sufficient for detecting subsequent mutation sites (Table S6).

### Genetic variant discrepancies in WES data

**Figure 1:**
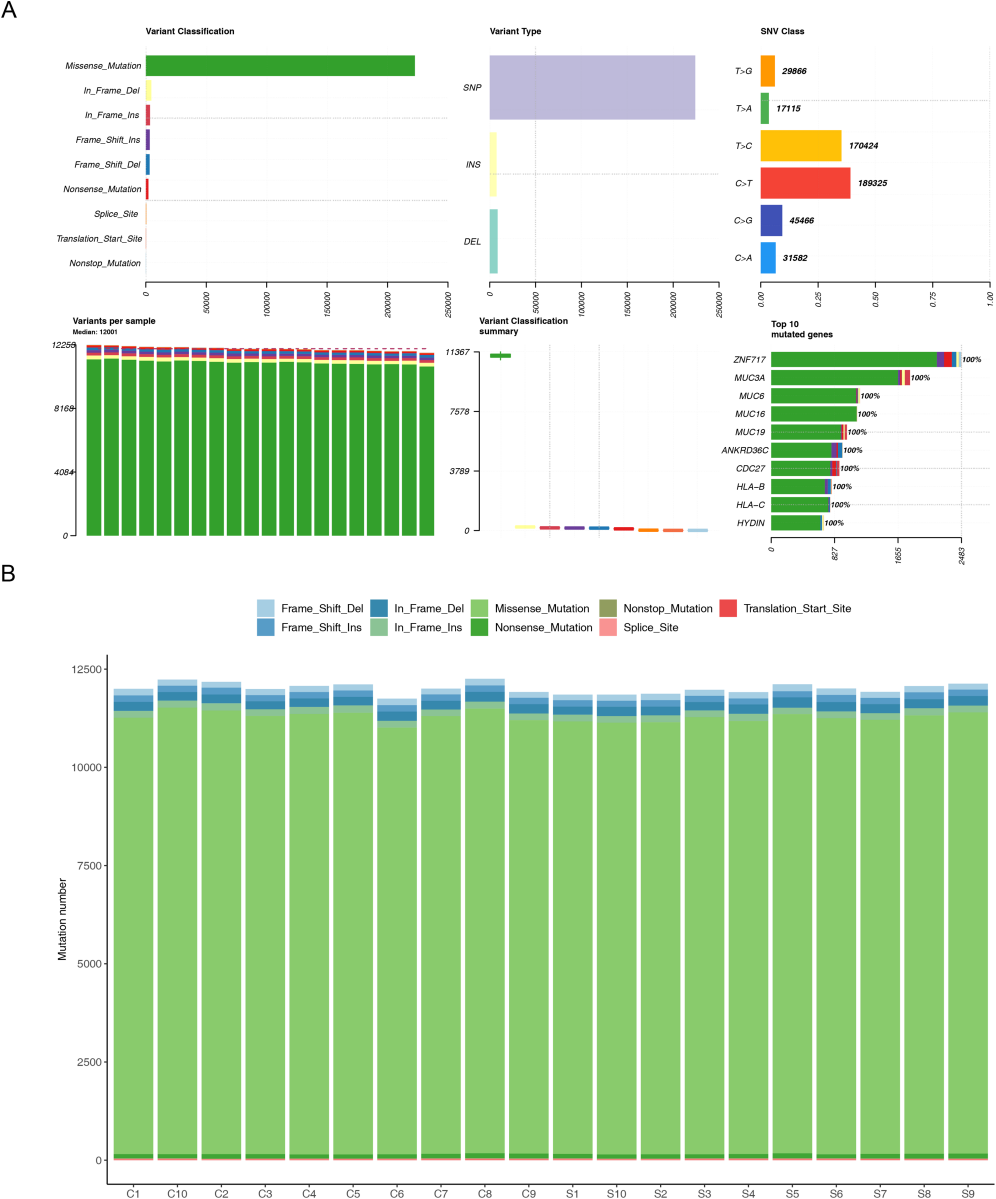
Discrepancies in somatic mutation frequencies as revealed by WES data. (A) Variant Classification Green: Missense Mutation; Yellow: In Frame Del: In Frame Deletion; Dark Red: In Frame Ins: In frame insertion; Purple: Frame Shift Ins: Frame shift insertion; Blue: Frame Shift Del: Frame shift deletion; Light red: Nonsense Mutation: Nonsense mutation; Orange: Splice Site: Splice site; Light Pink: Translation Start Site: Translation start site; Light Blue: Nonstop Mutation: Nonstop mutation Variant Type Lavender: SNP: Single nucleotide polymorphism; Light yellow: INS: Insertion; Pale blue: DEL: Deletion SNV Class Orange: T>G, Quantity: 29,886; Green: T>A: Quantity: 17,715; Yellow: T>C: Quantity: 170,424; Red: C>T: Quantity: 189,325; Dark blue: C>G: Quantity: 45,466; Light blue: C>A: Quantity: 31,582 Variants per sample: The green bars represent the number of variants. The specific values for different samples are marked above the bars. Variant Classification summary: Different colored lines (corresponding to the colors in “Variant Classification”) represent the summary of different variant classifications. Top 10 mutated genes: Different genes correspond to bars of different colors. The differently—colored regions within the bars represent the proportion of different variant classifications within that gene. The specific gene names and proportions are shown in the figure. Figure B Mutation type Light blue: Frame Shift Del: Frame shift deletion; Dark blue: In Frame Del: In-frame deletion; Green: Missense_Mutation: Missense mutation; Brown: Nonstop_Mutation: Non-stop mutation; Red: Translation Start Site: Translation start site; Blue: Frame Shift Ins: Frame shift insertion; Light green: In Frame Ins: In frame insertion; Dark green: Nonsense Mutation: Nonsense mutation; Pink: Splice Site: Splice site.

An overall examination of the WES data indicated that SNPs and missense mutations were the most frequent mutation types (Fig. 1A). Among all mutated genes, *ZNF717*, *MUC3A*, and *MUC6* were the top three in terms of the number of mutations (Fig. 1A). As presented in Fig. 1B, missense mutations were the most prevalent mutation type across the 20 specimens.

### The mutation patterns of HL patients

**Figure 2:**
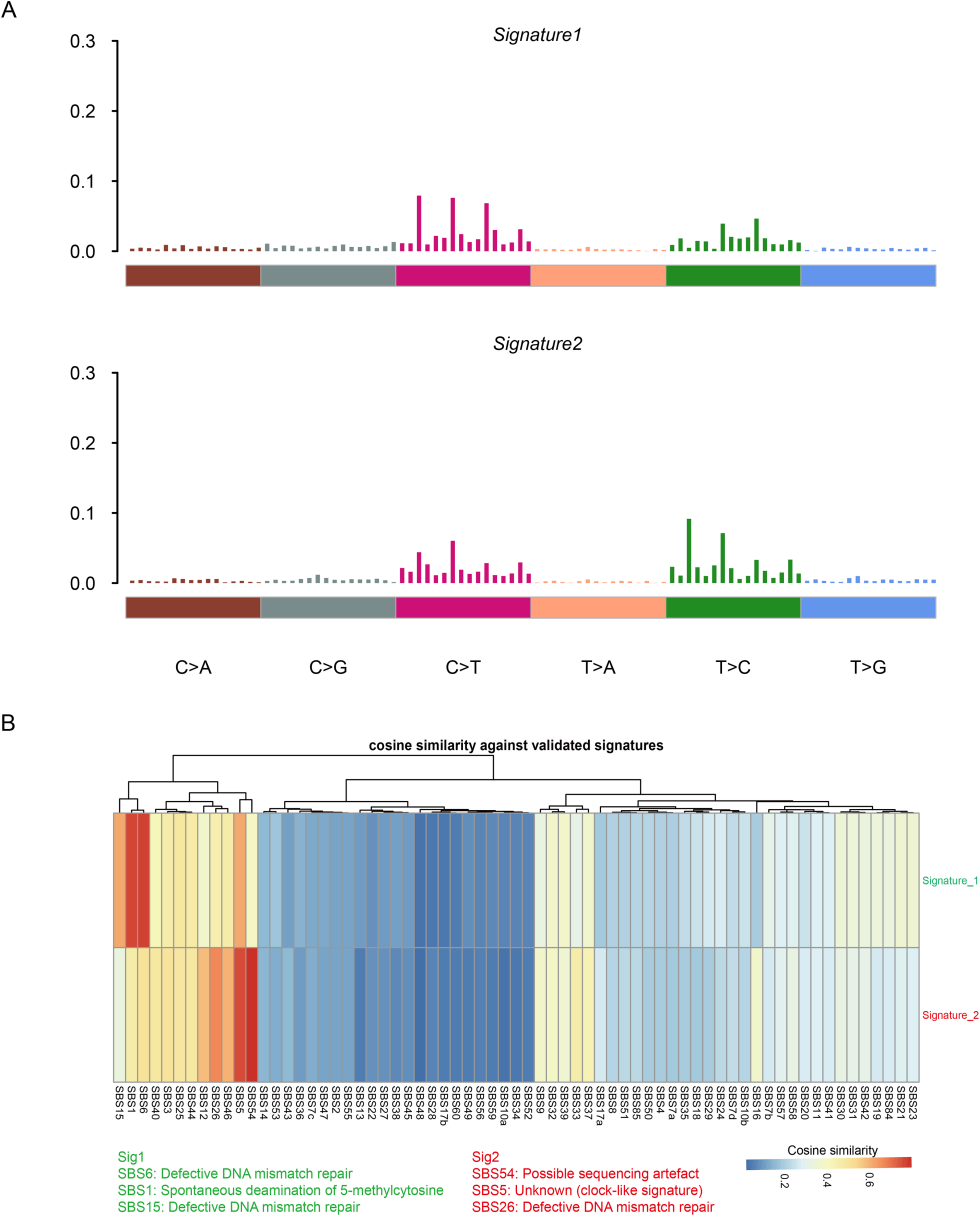
Identification of two mutation patterns among HL patients using copheneticanalysis. (A) Mutation Signatures Signature1: Presents the distribution of the frequencies of different base mutation types. The frequency values are shown in a bar—chart form, and the colored horizontal bar below represents different base mutation types. Signature2: Presents the distribution of the frequencies of different base mutation types. The frequency values are shown in a bar—chart form, and the colored horizontal bar below represents different base mutation types. Brown: C>A; Gray: C>G; Fuchsia: C>T; Orange: T>A; Green T>C; Blue: T>G (B); Validated Signatures Green: Sig1 (SBS1: Spontaneous deamination of 5-methylcytosine, SBS15: Defective DNA mismatch repair) Red: Sig2 (SBS54: Possible sequencing artefact, SBS55: Unknown (clock–like signature), SBS26: Defective DNA mismatch repair) Cosine Similarity: Different shades of blue are used to represent cosine similarity values. The darker the color, the higher the similarity; the lighter the color, the lower the similarity. The vertical bars of different colors represent the cosine similarity of different samples to the validated mutation signatures.

**Figure 3:**
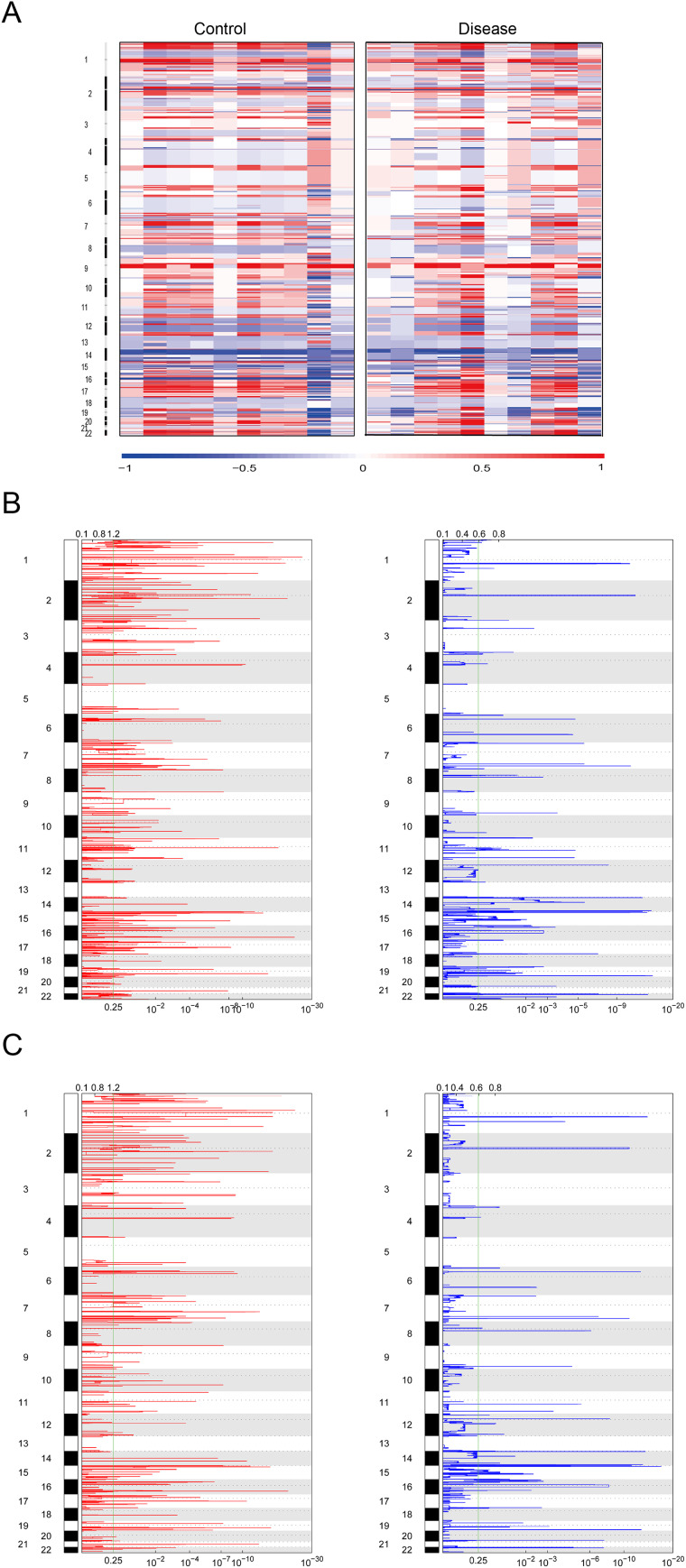
Mutation patterns in HL patients. (A) Sample Types Control: The left part of the heatmap shows the copy number variation (CNV) of chromosomes in the control group. Disease: The right part of the heatmap shows the copy number variation (CNV) of chromosomes in the disease group. Color Coding; Blue areas represent lower copy number variation values (such as −1). Red areas represent higher copy number variation values (such as 1). The color gradient reflects the transition from low to high copy number variation values. (B, C) Chromosome Position: The numbers on the vertical axis represent different chromosome numbers, indicating the position of variations on chromosomes. Copy Number Change Red lines indicate significant copy number amplifications. Blue lines indicate significant copy number deletions. Value Scale: The scales on the horizontal axis (such as 10^−2^, 10^−4^, *etc*.) are used to measure the degree of copy number change or relevant statistical values.

Cophenetic analysis revealed two mutation patterns (signature 1 and signature 2) among HL patients. The frequency of base mutations for these two signatures is displayed in Fig. 2A. Comparison with mutation signatures v3 from the COSMIC database showed that signature 1 exhibited high cosine similarity with SBS6, SBS15, and SBS1 (cosine similarity > 0.6), which are linked to defective DNA mismatch repair and spontaneous deamination of 5-methylcytosine. Signature 2, in contrast, showed strong similarity with SBS54, SBS5, and SBS26 (cosine similarity > 0.6), which are associated with sequencing artifacts and defective DNA mismatch repair (Fig. 2B). Additionally, chromosomal CNV analysis in HL and NC specimens is presented in Fig. 3A, with significant regions of copy number amplification (red) or deletion (blue) observed in both HL and NC specimens (Figs. 3B, 3C).

### Identification of hub genes for HL specimens

**Figure 4:**
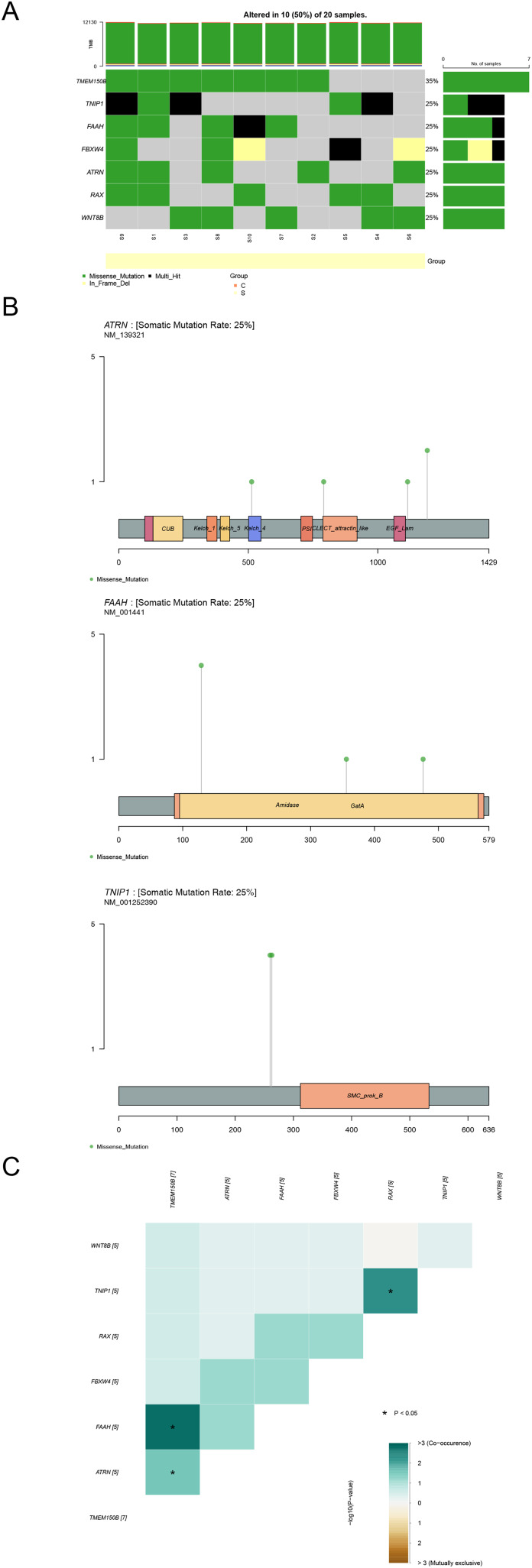
Identification of hub genes for HL specimen characterization. (A) Gene Mutation Status Green squares: Indicate that the gene has a mutation, and the mutation occurs in 10 out of 20 samples (50%). Gray squares: Indicate that the gene has no mutation. Black squares: Represent specific types of mutations (such as Missense_Mutation, In Frame Del). Yellow squares: Represent other specific mutation types (such as Mul_HH). Mutation Type Identification Missense_Mutation: Missense mutation (marked in black) In Frame Del: In frame deletion (marked in black) Mul HH: Specific mutation type (marked in yellow) Sample Groups “Group” is labeled above the horizontal axis. Different colors (such as red “C”, yellow “G”) represent different sample groups. (B) Gene Names The mutation status of A TRN1, *FAAH*, and *TNIP1* genes are shown respectively. Mutation Sites Different-colored regions on the gene sequence bar represent different types of mutation sites (such as Missense_Mutation). Vertical lines indicate the positions of mutation sites on the gene sequence, and green dots at the top of the vertical lines indicate the presence of mutations at these sites. (C) Gene Interaction Status Green squares of varying shades: Represent the co-occurrence of synergistic patterns among mutant genes. Darker colors indicate a higher number of co-occurrences, while lighter colors indicate a lower number of co-occurrences. Black asterisks (*): Indicate significant associations (*P* < 0.05) between corresponding gene pairs, such as those between *FAAH* and *TMEM150B*, *TNIP1* and *RAX*, and *TMEM150B* and *ATRN*.

Seven hub genes (*TMEM150B*, *TNIP1*, *ATRN*, *FAAH*, *FBXW4*, *RAX*, and *WNT8B*) were identified in HL. Most of these genes are specifically expressed in digestive organs such as the intestine, liver, and biliary system, and are involved in processes such as homeostasis/metabolism and immune system regulation (Table 2). Notably, *TMEM150B*, *TNIP1*, *ATRN*, and *FBXW4* were associated with immune functions, while *WNT8B* was linked to hepatocellular carcinoma (HCC) (Table 2). Mutation data for the seven hub genes are presented in Fig. 4A, with *TMEM150B* exhibiting the highest mutation frequency. The mutation sites of the hub genes are shown in Fig. 4B and Fig. S1. Co-occurrence analysis revealed significant associations between *FAAH* and *TMEM150B*, between *TNIP1* and *RAX*, and between *TMEM150B* and *ATRN* (*p* < 0.05) (Fig. 4C), suggesting a synergistic pattern in the alteration of these mutant genes. These findings support the hypothesis that concurrent mutations in these genes contribute to the development of HL complicated by hepatatrophy.

**Table 2:** Seven hub genes of HL’s mutation. Gene: Names of hub genes, including *TMEM150B*, *TNIP1*, *ATRN*, *FAAH*, *FBXW4*, *RAX*, and *WNT8B*. Number of mutation: The number of mutations in the gene. Function: Gene function, describing the mechanism of action of the protein encoded by the gene or the biological processes it is involved in. Main phenotype: Main phenotype, referring to the major physiological phenotypic characteristics presented due to the influence of gene—related functions. Expression: Gene expression sites, indicating in which tissues or organs the gene is expressed.

Gene	Number of mutation	Function	Main phenotype	Expression
*TMEM150B*	7	Encode a protein that belongs to the DRAM (damage-regulated autophagy modulator) family of membrane-spanning proteins.	Liver/biliary system phenotype, immune system phenotype	Colon duodenum spleen
*TNIP1*	5	Encode an A20-binding protein which plays a role in autoimmunity and tissue homeostasis through the regulation of nuclear factor kappa-B activation	Liver/biliary system phenotype, digestive/alimentary phenotype,endocrine/exocrine gland phenotype, immune system phenotype	Gallbladder duodenum spleen
*ATRN*	5	Encode a secreted protein that is involved in the initial immune cell clustering during inflammatory responses that may regulate the chemotactic activity of chemokines	Cardiovascular system phenotype,homeostasis/metabolism phenotype, endocrine/exocrine gland phenotype	Liver spleen
*FAAH*	5	Fatty acid amide hydrolase	Mortality/aging,homeostasis/metabolism phenotype	Kidney liver
*FBXW4*	5	A member of the F-box/WD-40 gene family and is used for ubiquitin-mediated degradation, its related pathways including Class I MHC mediated antigen processing and presentation		Liver kidney pancreas
*RAX*	5	Encode a homeobox-containing transcription factor that functions in eye development.		Cranial nerve,neuroretina
*WNT8B*	5	A member of the WNT gene family and is associated with hepatocellular carcinoma	Nervous system phenotype	Nervous system kidney-cortical intestine

### Exploration of the potential functions of hub genes

**Figure 5:**
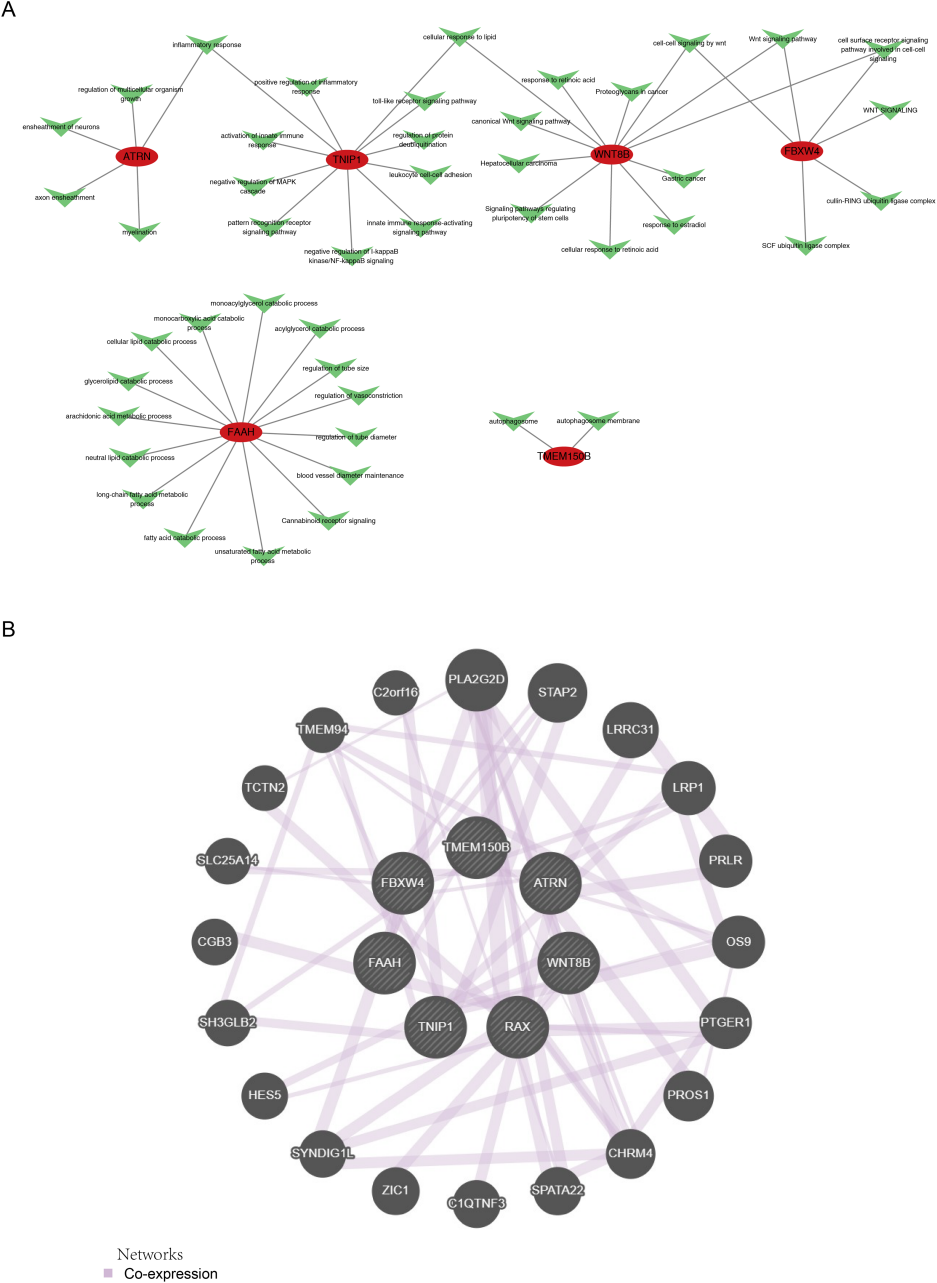
Regulatory mechanisms and correlations among hub genes. (A) Gene Nodes Red oval nodes: Represent hub genes. Green triangular nodes: Represent genes or elements in enriched pathways. Lines: Grey lines indicate the regulatory relationships between hub genes and genes or elements in enriched pathways. (B) Gene Nodes: Dark grey circular nodes represent genes, including seven hub genes and 20 co—expressed genes. Some gene names are labeled on the nodes, such as PLA 2 G2D, STA P 2, *etc*. Light purple lines (Networks): Indicate co—expression relationships between genes.

To explore the potential pathways associated with hub genes, GO and KEGG enrichment analyses were conducted. The GO analysis revealed that *FBXW4* and *WNT8B* were linked to pathways involved in cellular response to lipids, Wnt signaling, cell-surface receptors, and cell-cell signaling, while *ATRN* and *TNIP1* were associated with inflammatory responses (Table S7). KEGG analysis identified enrichment in pathways regulating pluripotency of stem cells and Wnt signaling for *WNT8B* (Table S7). Additionally, the regulatory relationships between hub genes and the enriched pathways are illustrated in Fig. 5A.

### The network of PPI, ceRNA, TF-mRNA, and TF-ceRNA

**Figure 6:**
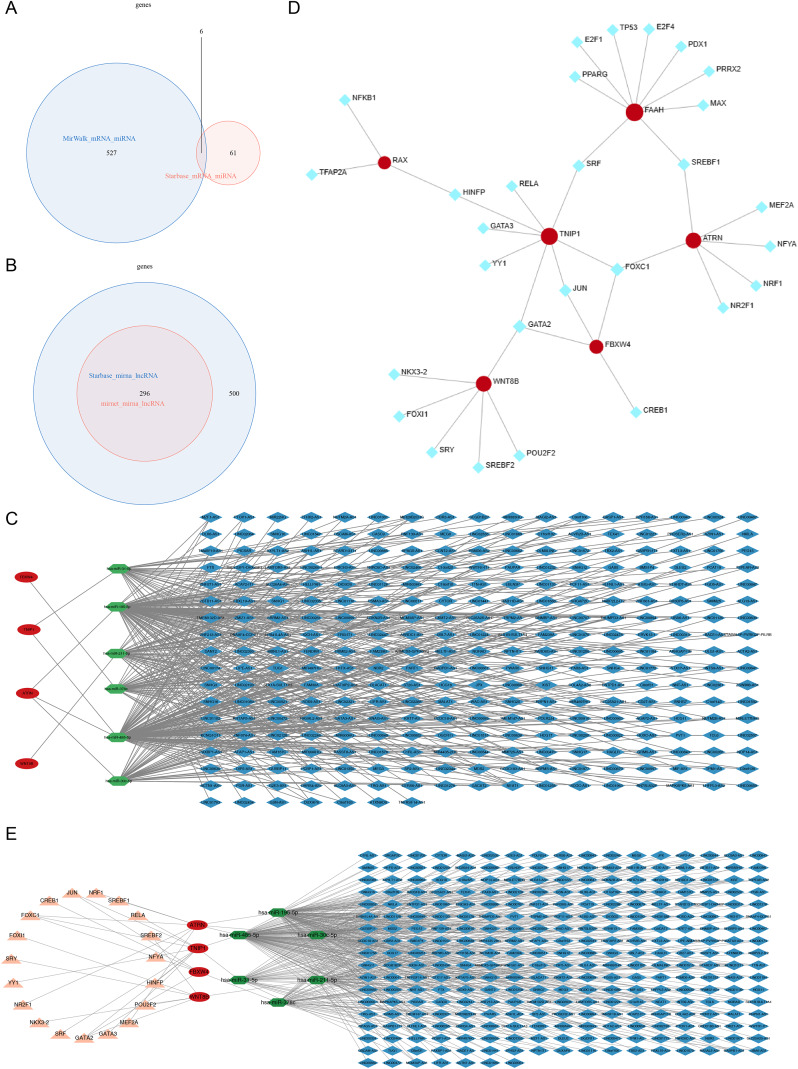
Regulatory mechanisms of seven hub genes identified through miRNAs andlncRNAs. (A) The overlapping part of the two ovals: Represents the common miRNAs in the two databases, with a quantity of six. Target miRNAs: This figure aims to show the six miRNAs targeting hub genes identified through the two databases. (B) The overlapping part of the two ovals: Represents the common lncRNAs in the two databases, with a quantity of 296. Target lncRNAs: This figure shows the 296 lncRNAs targeting 6 miRNAs identified through the two databases. (C) Gene and RNA Nodes Red dots: Represent genes (such as LINC01128, TNFRSF14-AS1, SNHG12). Green dots: Represent miRNAs (such as hsa-miR-195-5p). Lines: Gray lines indicate the regulatory relationships between genes and miRNAs. (D) Gene and Transcription Factor Nodes Red dots: Represent 6 hub genes. Light blue dots: Represent 28 transcription factors (TFs). Lines: Gray lines indicate the regulatory relationships between hub genes and transcription factors, constructing the TF-mRNA network. (E) Gene, RNA, and Transcription Factor Nodes Red triangles: Represent 4 hub genes. Green dots: Represent 6 miRNAs. Red dots: Represent 19 transcription factors (TFs). Light blue diamonds: Represent 296 lncRNAs. Lines: Gray lines indicate the regulatory relationships among different types of molecules, constructing the ceRNA network.

To investigate potential correlations among hub genes, a protein-protein interaction (PPI) network comprising the seven hub genes and 20 co-expression genes was constructed (Fig. 5B). To further understand the regulatory mechanisms of these genes, miRNAs and lncRNAs targeting the hub genes were explored. Six miRNAs targeting the hub genes were identified from the miRWalk and Starbase databases (Fig. 6A). Subsequently, 296 lncRNAs targeting these six miRNAs were obtained from the MiRNet and Starbase databases (Fig. 6B). A competing endogenous RNA (ceRNA) network, consisting of 306 nodes and 415 edges, was established based on the six miRNAs, four hub genes, and 296 lncRNAs (Fig. 6C). This network revealed that LINC01128, TNFRSF14-AS1, and SNHG12 could bind to hsa-miR-195-5p, thereby regulating *ATRN*. Moreover, 28 TFs targeting the hub genes were identified. A TF-mRNA network (34 nodes and 36 edges) was created based on the six hub genes and 28 TFs (Fig. 6D), which demonstrated that SREBF1 could regulate the transcription of *ATRN*, *FAAH*, and *WNT8B*. Additionally, a TF-ceRNA network (325 nodes and 439 edges) was constructed based on the four hub genes, six miRNAs, 19 TFs, and 296 lncRNAs (Fig. 6E).

### Prediction of potential drugs for HL

**Figure 7:**
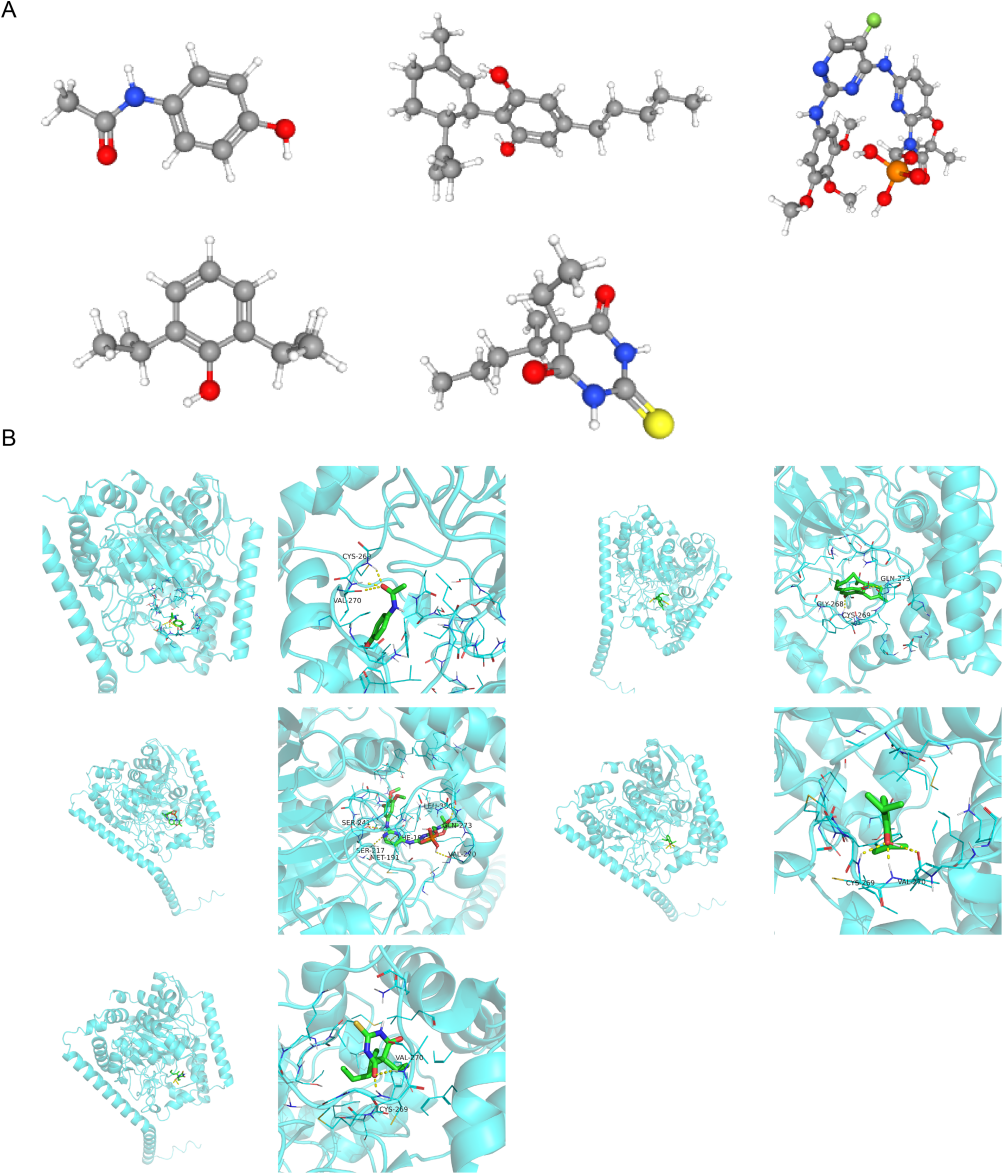
(A) Drug structures: displays the corresponding structures of five drugs retrieved from the DrugBank database. Different molecular structure models distinguish atom types by color. For example, gray represents carbon atoms, red represents oxygen atoms, blue represents nitrogen atoms, *etc*., presenting the three dimensional spatial structure information of the drugs. (B) Protein—Drug Docking Results Protein Structure: Presents the protein structure of *FAAH* (fatty acid amide hydrolase) as a light green cartoon model, reflecting its secondary structure features (such as α-helices, β-sheets, *etc*.). Drug Molecules: Presents different drugs (thiopental, propofol, cannabidiol, fostamatinib, acetaminophen) as stick models in colors like green and yellow, showing their binding at the active sites of the *FAAH* protein and the spatial positional relationships of drug—protein interactions. B-1 to B-5: Represent the molecular docking results of different drugs with the *FAAH* protein, presenting the details of the binding modes from different perspectives.

To identify potential therapeutic drugs for HL patients with hepatatrophy, drug predictions were performed. A total of five drugs targeting *FAAH* were retrieved from the DrugBank database (Table 3), and their corresponding structures are shown in Fig. 7A. Molecular docking results for *FAAH* with thiopental, propofol, cannabidiol, fostamatinib, and acetaminophen are displayed in Fig. 7B. Additionally, Table 4 shows that fostamatinib exhibited the best binding affinity with *FAAH*, with a binding energy of −9.2 kcal/mol. Nonetheless, this finding requires further experimental validation.

**Table 3:** Five drugs from DrugBank database to target *FAAH*. Drug: Drug names, including Thiopental, Propofol, Cannabidiol, Fostamatinib, and Acetaminophen. gene: The gene targeted by the drug, all being the *FAAH* gene. FDA: The approval status of the drug by the U.S. Food and Drug Administration (FDA), all being “approved”. drug id: The identification number of the drug in the DrugBank database, which are DB00599, DB00818, DB09061, DB12010, and DB00316 respectively.

Drug	Gene	FDA	Drug_id
Thiopental	*FAAH*	Approved	DB00599
Propofol	*FAAH*	Approved	DB00818
Cannabidiol	*FAAH*	Approved	DB09061
Fostamatinib	*FAAH*	Approved	DB12010
Acetaminophen	*FAAH*	Approved	DB00316

**Table 4:** The binding energy of *FAAH* with fostamatinib. Drug: Drug names, including Thiopental, Propofol, Cannabidiol, Fostamatinib, and Acetaminophen. Binding Energy (kcal/mol): The binding energy between the drug and the *FAAH* protein, with the unit of kcal/mol. The value reflects the tightness of the binding between the drug and *FAAH*. A negative value indicates that energy is released during the binding process, and the larger the absolute value, the tighter the binding.

Drug	Binding energy (kcal/mol)
Thiopental	−6.5
Propofol	−6.7
Cannabidiol	−6.5
Fostamatinib	−9.2
Acetaminophen	−6.6

### Molecular dynamics validation of *FAAH*

**Figure 8:**
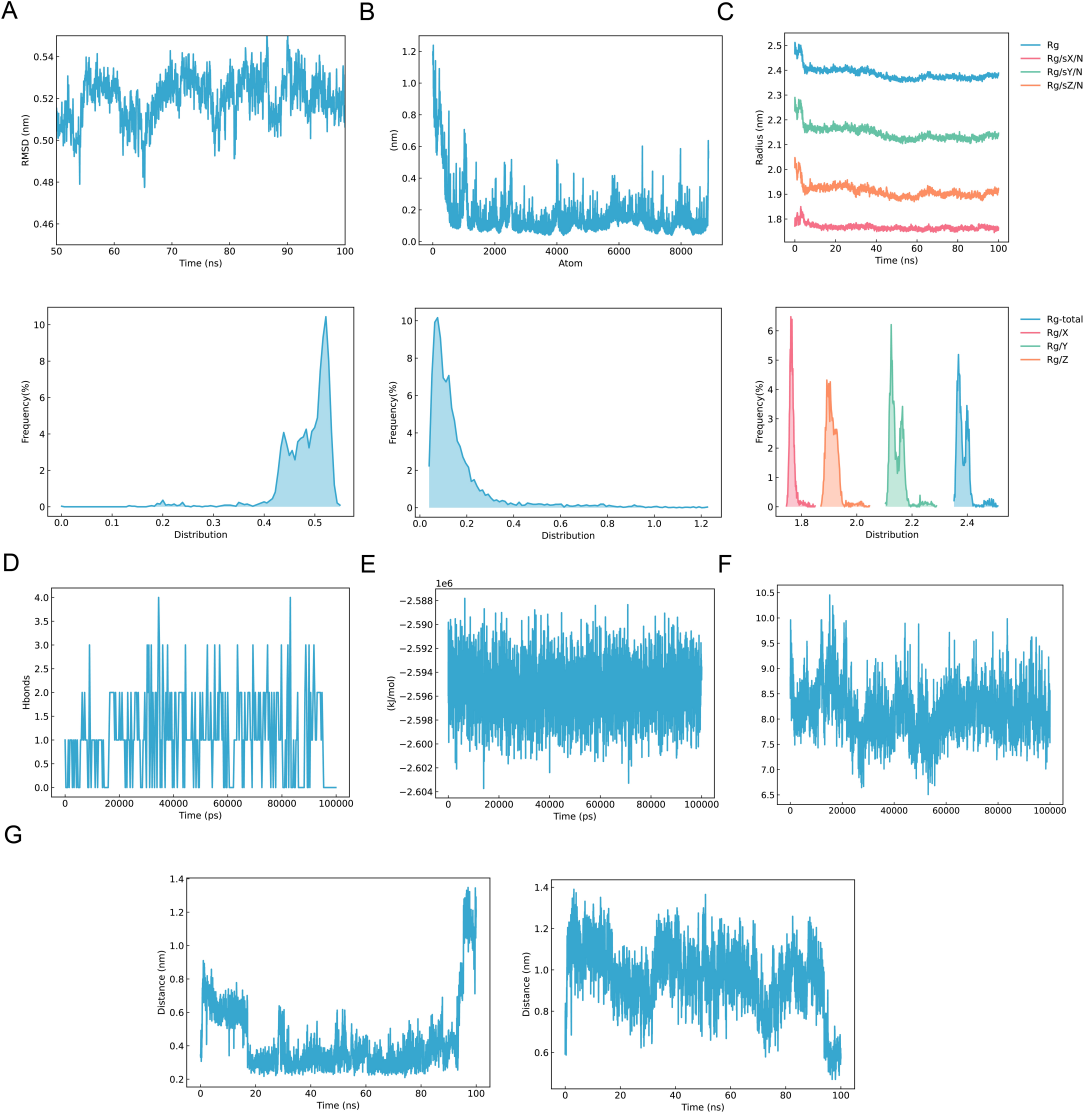
Molecular dynamics validation of *FAAH* (100 ns). (A) RMSD plot of protein *FAAH*. (B) RMSF plot of protein *FAAH*. (C) Radius of gyration plot for protein *FAAH*. (D) Plot of the number of hydrogen bonds between small molecule drug and the active site of protein. (E) Bond energy diagram of small molecule drug and protein. (F) Area map of the interface between small molecule drug and protein. (G) Plot of H-atom distances of small molecule drugs to *FAAH* amino acid residues.

A 100 ns MD simulation was performed on *FAAH*. The results revealed that the RMSD of *FAAH* remained stable, fluctuating between 0.49 and 0.55, indicating that the protein reached a stable state from 50 to 100 ns (Fig. 8A). The RMSF value of *FAAH* ranged between 0.1 and 0.5, indicating good flexibility of the protein amino acids and stable binding with the small molecule drug ligands throughout the simulation (Fig. 8B). The Rg value of *FAAH* fluctuated around 2.4 nm, suggesting that the protein peptide chain remained in a stable conformation during the simulation (Fig. 8C). Additionally, the number of hydrogen bonds between the small molecule drug and the active site remained stable, typically ranging from 1 to 3, with occasional fluctuations up to 4 (Fig. 8D). These fluctuations were likely caused by intermittent interference from water molecules. Overall, the small molecule maintained stable binding with the active site throughout the simulation. The bond energy remained consistent, within the range of −2.602 to −2.588 kJ/mol, indicating stable bond interactions between the protein and ligand, and a robust complex structure (Fig. 8E). The interfacial area between the protein and small molecule ligand also remained stable, fluctuating between 7–10 Å^2^, reflecting the stability of the binding affinity and the interaction range (Fig. 8F). Finally, upon binding, the highly electronegative F atom formed covalent interactions with the H atom on glycine at position 268 and the H atom on leucine at position 278. The distance between the F atom and the glycine H atom fluctuated between 0.2 and 0.7 nm, while the distance between the F atom and the leucine H atom ranged from 0.6 to 1.4 nm, indicating stable binding interactions (Fig. 8G).

### Molecular dynamics simulation of 80 ns

**Figure 9:**
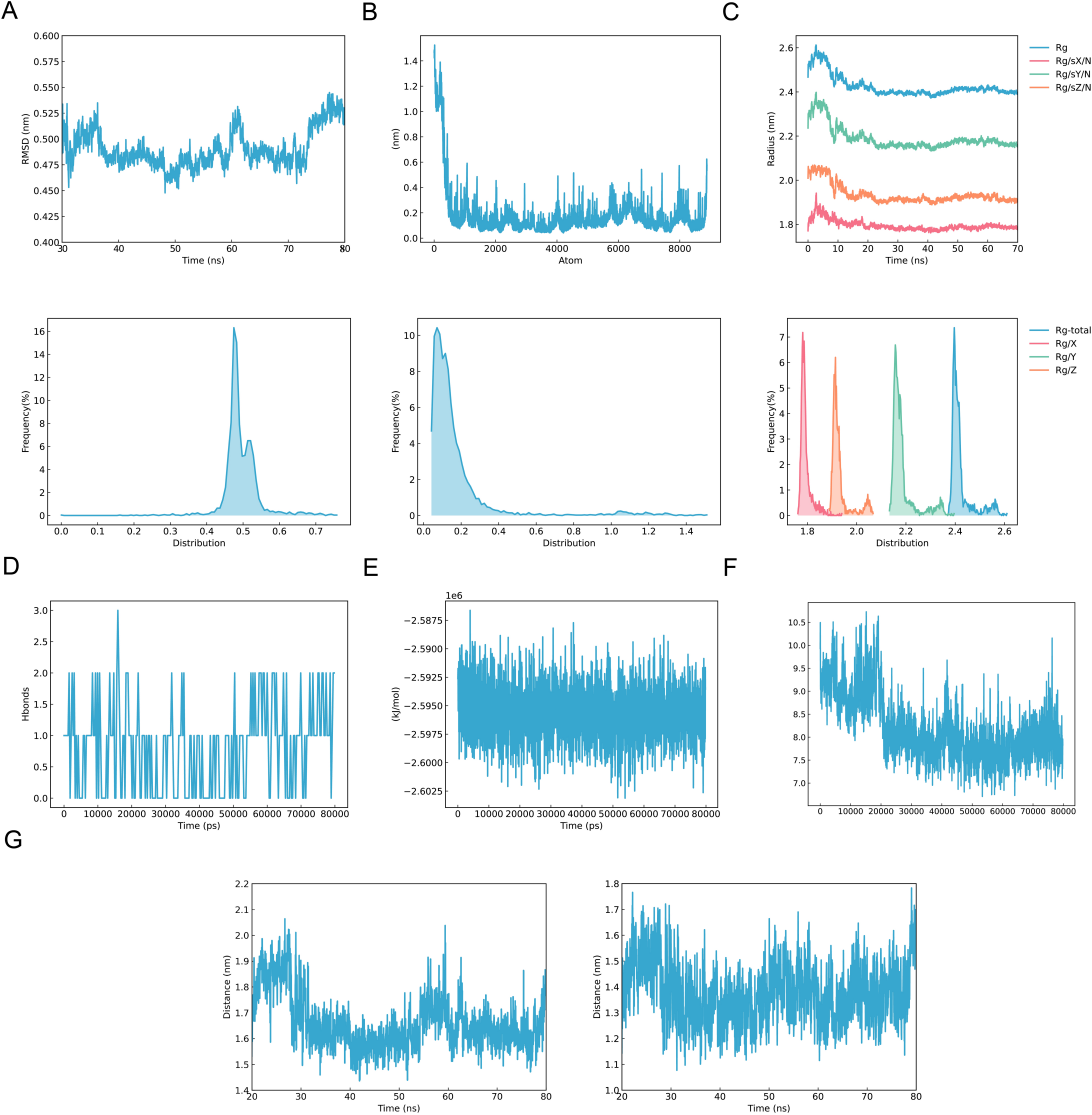
Molecular dynamics validation of *FAAH* (80 ns). (A) RMSD plot of protein *FAAH*. (B) RMSF plot of protein *FAAH*. (C) Radius of gyration plot for protein *FAAH*. (D) Plot of the number of hydrogen bonds between small molecule drug and the active site of protein. (E) Bond energy diagram of small molecule drug and protein. (F) Area map of the interface between small molecule drug and protein. (G) Plot of H-atom distances of small molecules.

To ensure the reproducibility of the results, an 80 ns MD simulation of *FAAH* was conducted. The RMSD of *FAAH* remained between 0.45 and 0.55, indicating protein stability from 30 to 80 ns (Fig. 9A). The RMSF ranged from 0.1 to 0.6, suggesting good flexibility of the protein’s amino acids and stable interactions with the small molecule drug ligands throughout the simulation (Fig. 9B). The Rg value fluctuated around 2.4 nm, confirming that the protein’s peptide chain remained stable during the entire simulation (Fig. 9C). Additionally, the number of hydrogen bonds between the small molecule and the active site remained mostly stable, varying between 1 and 2, with occasional increases to 3 (Fig. 9D). These fluctuations were likely caused by brief interference from water molecules. Consequently, the small molecule maintained stable binding with the active site throughout the simulation. The bond energy plot demonstrated stability within the range of −2.602 to −2.588 kJ/mol, confirming that the protein-ligand interactions were stable and the complex structure robust (Fig. 9E). Furthermore, the interfacial area between the protein and ligand remained steady, fluctuating between 6.5 and 10 Å^2^, indicating consistent binding strength and interaction range (Fig. 9F). Finally, during protein-ligand binding, the highly electronegative F atom formed covalent interactions with the H atoms of glycine at position 268 and leucine at position 278. The distance between the F atom and the glycine H atom ranged from 1.4 to 2 nm, while the distance between the F atom and the leucine H atom fluctuated between 1.1 and 1.7 nm, indicating stable binding (Fig. 9G).

### Hub genes were confirmed by RT–qPCR

**Figure 10:**
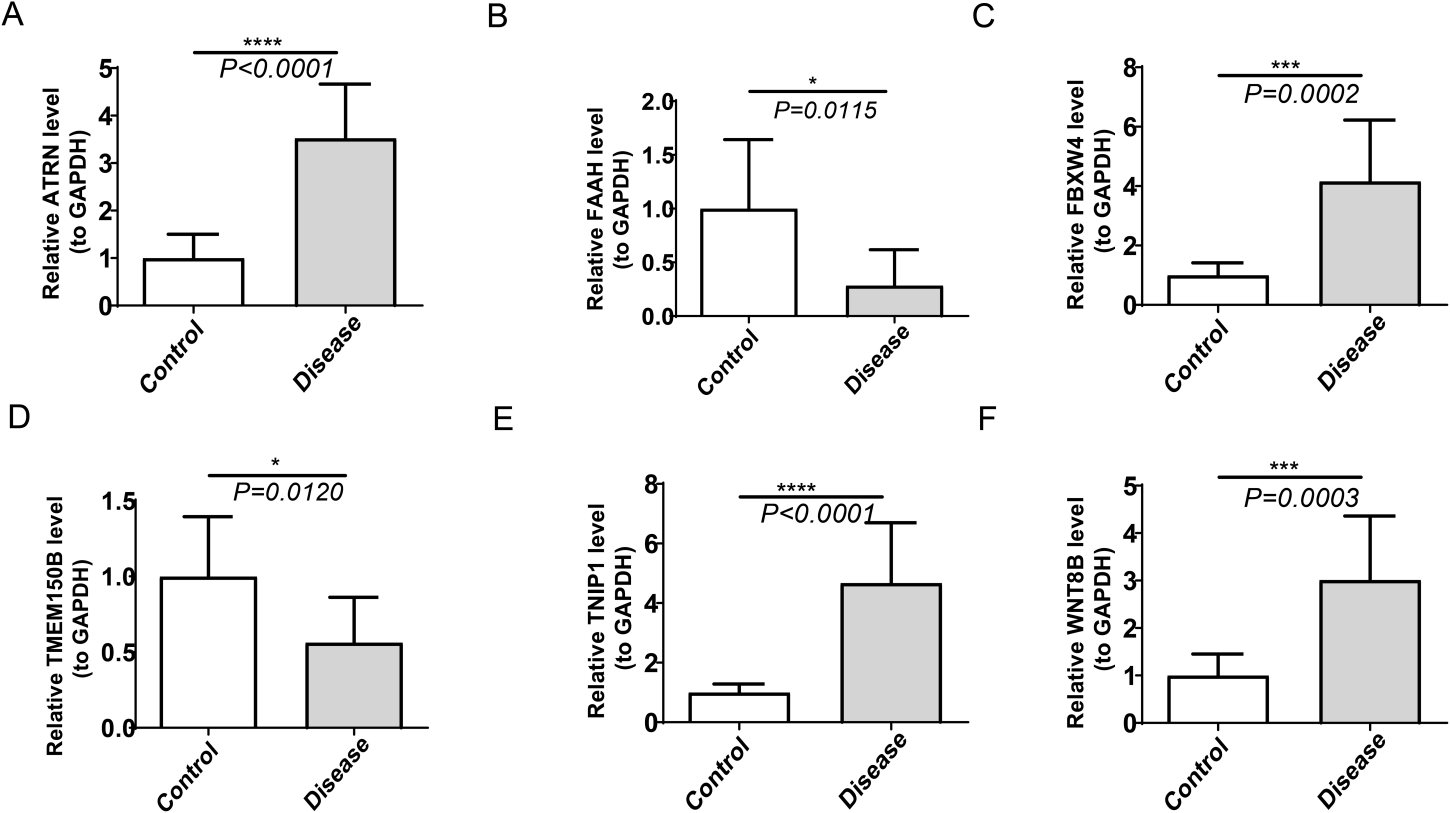
Expression levels of RT-qPCR results in the HL group compared to the normal group. (A) *ATRN* (B) *FAAH* (C) *FBXW4* (D) *TMEM150B* (E) *TNIP1* (F) *WNT8B*. * indicates *p*-value < 0.05, *** indicates *p*-value < 0.001, **** indicates *p*-value < 0.0001.

RT–qPCR results indicated that the expression levels of *TMEM150B* and *FAAH* were significantly lower in the HL group compared to the normal group, while the expression levels of *TNIP1*, *ATRN*, *FBXW4*, and *WNT8B* were significantly higher in the HL group compared to the normal group (Fig. 10). However, reliable amplification for *RAX* was not achieved under our experimental conditions, precluding the determination of its expression level.

## Discussion

HL is rare but prevalent in East Asia (Azhdari et al., 2022). It forms in the liver’s bile duct system and consists of cholesterol, bile salts, or calcium salts. Treatment is challenging due to the complex anatomy and diverse shapes of the ducts. While traditional surgeries such as open or laparoscopic methods are effective, they carry risks and involve long recovery times (Ito, Warnken & May, 1999; Le Guerroué et al., 2023). Furthermore, many patients experience recurrent HL and atrophy of the intrahepatic bile ducts postoperatively. In some cases, persistent stone recurrence and chronic inflammation lead to malignant transformation, creating a vicious cycle that represents a major challenge in managing this disease. Therefore, elucidating the pathogenesis of intrahepatic bile duct fibrosis induced by HL is of paramount importance. WES can reveal the mutation spectrum of diseases, offering new approaches for understanding disease mechanisms, diagnosis, and treatment (Restuadi et al., 2022). In the present study, WES was performed on peripheral blood samples from both normal individuals and patients with HL-related liver atrophy, providing comprehensive genomic information and identifying seven high-frequency core mutated genes, including TMEM150B, TNIP1, ATRN, FAAH, FBXW4, RAX, and WNT8B. This highlights the potential significance of molecular targeting for specific biological pathways.

Transmembrane protein 150B (TMEM150B) exhibited the highest mutation frequency in this study. Additionally, TMEM150B promotes cell survival under stress conditions by regulating autophagy (Abouzeid et al., 2012; Bennett et al., 2008). Variations in the TMEM150B gene locus have been linked to early menopause in females (Li et al., 2021). Additionally, the TMEM150B SNP rs11668344 may be associated with reduced involution of terminal ductal lobular units (TDLUs) and an increased risk of early menopause (Tokuda, Kuramoto & Serikawa, 2004). However, the role of TMEM150B in the liver, particularly its function in chronic inflammation and fibrosis, remains unexplored. Based on existing research, it is speculated that TMEM150B may contribute to the development of HL-related liver atrophy through mutation at a specific SNP locus.

TNFAIP3-Interacting Protein 1 (TNIP1) is a nuclear factor kappa-B (NF-κB) regulator, playing a pivotal role in immune diseases and inflammatory responses (Shahrour et al., 2017). Over the past decades, TNIP1 has been identified as a strong risk locus for various autoimmune diseases, with significant associations to systemic lupus erythematosus, certain clinical features, and the regulation of mitochondrial autophagy (Farooqui, Elhence & Shalimar, 2022; Lu et al., 1999). Notably, TNIP1 has also been implicated in the pathogenesis of autoimmune hepatitis and viral hepatitis. The underlying mechanisms may involve the regulation of mitophagy and the NF-κB signaling pathway, influencing the balance between hepatocyte repair and apoptosis (Cheng et al., 2019; Lei et al., 2020; Merline & Schaefer, 2023; Oka et al., 2018). This study found mutations in TNIP1 in all five HL patients, with two mutation sites present in four of these patients, indicating double mutations in TNIP1. Moreover, TNIP1 was highly expressed in HL tissues, where it may exacerbate inflammatory responses and fibrosis by inhibiting hepatocyte autophagy, contributing to hepatic atrophy progression. These findings suggest that TNIP1 may play a dual regulatory role in HL-associated hepatic atrophy, involving immune regulation and autophagy modulation. This study represents the first identification of high-frequency TNIP1 gene mutations in HL cases, which may be linked to mutations in conserved motifs and structural domains, warranting further investigation.

Retinal homeobox (RAX) is an interferon-inducible activator of double-stranded RNA-dependent protein kinase (PKR) (Tessitore et al., 1989). Complete deletion of the RAX gene at the preimplantation stage in mice leads to embryonic lethality (Gandhi et al., 2002). However, there are relatively few reports of mutations in the human RAX gene (Thibaut et al., 2021). Our analysis revealed a high mutation frequency of RAX in HL, with co-occurrence alongside TNIP1, suggesting that these mutations may synergistically promote disease progression. Despite its importance in immune response, research on RAX in the liver is limited. Known to activate the PKR pathway (Bennett et al., 2012), RAX may be linked to hepatic inflammation and fibrosis. PKR, a key component of antiviral innate immunity, amplifies pro-inflammatory cytokines and induces apoptosis when dysregulated, contributing to the pathogenesis of autoimmune hepatitis and viral hepatitis (Balachandran & Barber, 2007; Chen & Harris, 2023). Based on this, RAX mutations may disrupt the immunoregulatory axis, potentially driving a persistent interferon response in HL, thereby sustaining a chronic inflammatory environment in the liver.

This study identified Attractin (ATRN) as a novel mutant gene in HL. While ATRN has traditionally been linked to neuropsychiatric disorders and myelination in the central nervous system due to its role in mitigating oxidative stress and inhibiting apoptosis (Bari et al., 2023; Lei et al., 2022; Rafiei & Kolla, 2021), its function in the liver is increasingly recognized. Recently, ATRN has been characterized as an immunomodulatory protein (Liu et al., 2023). It is hypothesized that ATRN mutations contribute to the pathogenesis of HL and secondary hepatic atrophy by disrupting immune homeostasis in the liver and promoting a profibrotic microenvironment. The persistent biliary inflammation characteristic of HL subjects liver immune cells, including macrophages, to chronic stimulation (Vlăduţ et al., 2020). In this context, loss-of-function mutations in ATRN may impair its ability to modulate immune responses, triggering excessive or dysregulated inflammatory reactions. Chronic inflammation serves as a central driver for the activation of hepatic stellate cells (HSCs) and fibrogenesis (Tanwar et al., 2020). This fibrotic process, coupled with enhanced oxidative stress—a consequence of ATRN deficiency—can directly damage cholangiocytes and hepatocytes, disrupt bile acid homeostasis, and ultimately impair liver regeneration and cause atrophy (Allameh et al., 2023). Thus, ATRN functions as a key local regulator within the hepatic immune microenvironment. Mutations in ATRN may predispose the liver to more severe inflammatory and fibrotic responses upon biliary injury, accelerating disease progression from intrahepatic stones to hepatic atrophy. This novel finding positions ATRN at the intersection of three core pathological pathways in hepatobiliary diseases—immune dysregulation, oxidative stress, and fibrosis—offering a promising new target for mechanistic investigation.

Fatty acid amide hydrolase (FAAH) is an enzyme responsible for hydrolyzing the endogenous cannabinoid anandamide (AEA) (Lockwood et al., 2013). Alterations in the activity of this intracellular enzyme have been linked to various neuropsychiatric disorders, including major depressive disorder (Li, Angione & Milunsky, 2015). Inhibition of FAAH has been shown to exert analgesic and anti-inflammatory effects (Liu et al., 2021; Perugorria et al., 2019; Shree Harini & Ezhilarasan, 2023; Chen et al., 2014). Exon sequencing revealed FAAH mutations in five HL patients, with mutation site 1 present in four of these patients, indicating a commonality in FAAH mutations. RT–qPCR analysis further revealed low expression of FAAH in the HL group, suggesting that FAAH may serve as a potential biomarker in the treatment of HL. Moreover, the TF-mRNA network indicated that FAAH mutations may influence the regulation of upstream TFs, although the precise mechanism requires further investigation. Notably, the mutation sites at 1–30 amino acids in FAAH are predicted to reside in a transmembrane region, and loss of this site could lead to protein aggregation. FAAH’s serine nucleophile (Ser-241) forms an unusual catalytic triad with Ser-217 and Lys-142 (Liu et al., 2011). Mutation of these three sites to alanine significantly reduces FAAH’s catalytic activity. Additionally, mutations in other FAAH sites can also affect its enzyme activity (Mitani et al., 2017). While FAAH is primarily associated with neurological functions, it is also expressed in the liver, particularly in HSCs (Wojtalla et al., 2012). In the present study, reduced FAAH expression in HL was observed, which may lead to the accumulation of AEA (Della Pietra et al., 2023) and subsequently promote HSC apoptosis. This process may have dual effects in hepatic fibrosis: while it may contribute to fibrosis regression, the aberrant depletion of HSCs could disrupt hepatic microenvironmental homeostasis and regenerative capacity, thereby indirectly facilitating hepatocyte injury and atrophy progression.

F-box and WD repeat domain-containing 4 (FBXW4) is known to undergo mutation, loss, and low expression in various human cancer cell lines and clinical samples (Ianakiev et al., 1999). Chromosomal analysis using SNP chips detected a duplication in exon 9 near the FBXW4 gene in cases of split-hand/foot malformation (SHFM) (Saitoh, Mine & Katoh, 2002a; Saitoh, Mine & Katoh, 2002b). In this study, overexpression of FBXW4 was observed in the HL group via RT–qPCR, suggesting that it may be a risk factor for HL progression. However, the specific mutation type that leads to FBXW4 mutation in hepatobiliary atrophy with cholangiolithiasis remains to be further explored, providing a clear direction for future research.

Wnt family member 8B (WNT8B), a classical Wnt ligand, is associated with tumorigenesis and is a member of the Wnt family (Shochat et al., 2021). The present study is the first to report that WNT8B is mutated and tends to be overexpressed in the HL group, where it also regulates the signaling pathway governing stem cell pluripotency. Notably, GO results reveal significant enrichment of WNT8B and FBXW4 in the Wnt signaling pathway. Previous studies have shown that the Wnt/β-catenin signaling pathway plays a critical role in liver development, regeneration, metabolic zoning, ammonia and drug detoxification, and hepatobiliary development, maintaining liver homeostasis (Cheng et al., 2022). In two of the most common primary adult liver cancers—HCC and cholangiocarcinoma (CCA)—the Wnt/β-catenin pathway is often overactivated, promoting tumor cell proliferation, migration, and invasion (Paik, 2021). Liu et al. (2021) found that upregulation of WNT8B activates the canonical Wnt pathway, potentially further promoting liver cancer cell proliferation (Cheng et al., 2022). Furthermore, WNT8B is expressed in freshly isolated biliary epithelial cells and primary biliary epithelial cells, where it is associated with bile synthesis (Hoofnagle et al., 2013). Hiroyuki Mizuguchi and colleagues demonstrated that high levels of WNT8B secreted by hepatocytes and cholangiocytes can promote the transformation of hepatocyte-like cells (Matsukane et al., 2022). In the present study, the overexpression of WNT8B in HL may promote hepatic compensatory hyperplasia and fibrosis through the activation of the Wnt/β-catenin signaling pathway, contributing to the development of hepatobiliary calculi. However, the effect of WNT8B on β-catenin levels in the nucleus and cytoplasm requires further characterization through molecular experiments, such as Western blotting. Additionally, research has identified F-box/WD40 repeat proteins encoded by a novel human gene, Dactylin, which regulates development by specifically ubiquitinating and degrading target proteins in the Wnt and NF-κB signaling pathways (Bolger, Lohse & Usadel, 2014). In the present study, overexpressed FBXW4 may promote the development of hepatobiliary calculi through its effects on target proteins in the WNT signaling pathway, offering a new gene target for further exploration of FBXW4’s specific mechanisms in this disease, warranting continued investigation (Li & Durbin, 2009; Li et al., 2009b).

Sterol regulatory element-binding transcription factor 1 (SREBF1) regulates lipid homeostasis and is a key factor in lipid metabolism (DePristo et al., 2011). In non-alcoholic fatty liver disease (NAFLD), ApoA4 inhibits liver steatosis by suppressing SREBF1-mediated lipid synthesis, suggesting that SREBF1 is an important TF associated with obesity-related conditions (Mayakonda et al., 2018). Within the TF-mRNA network, SREBF1 regulates the transcription of ATRN, FAAH, and WNT8B. Clinically, patients with hepatic atrophy typically present with a frail phenotype. Mutations in these three core genes may lead SREBF1 to play a role in lipid synthesis pathways, contributing to the frail phenotype observed in these patients. However, the specific mechanistic processes require further study.

Expanding on our research, potential drugs targeting the core gene FAAH were predicted. Fostamatinib, the first spleen tyrosine kinase (Syk) inhibitor approved for treating adult chronic immune thrombocytopenia unresponsive to previous treatments, works by inhibiting Syk activation in macrophages, thereby blocking autoantibody-mediated platelet phagocytosis (Mermel et al., 2011; Shannon et al., 2003). HL indirectly leads to a decrease in platelet count in the peripheral blood. Fostamatinib is metabolized into its active component R406 in the intestine and primarily metabolized in the liver by cytochrome 3A4 and UGT1A9, suggesting potential therapeutic effects on the liver (da Silveira et al., 2019). FAAH plays a critical role in suppressing autoimmune inflammatory diseases (Perugorria et al., 2019). The present study observed reduced FAAH expression in HL, which may reflect a compensatory anti-inflammatory response under chronic inflammatory conditions (Tabas & Glass, 2013). Previous research has demonstrated that pharmacological modulation of this pathway can regulate endocannabinoid and inflammatory signaling, potentially offering therapeutic benefits in liver disease models, even with FAAH dysfunction (Bajaj et al., 2021; Della Pietra et al., 2023; van Egmond, Straub & van der Stelt, 2021). Based on these findings, it is proposed that fostamatinib may inhibit Syk-mediated sustained inflammation, reduce AEA depletion, and alleviate oxidative stress, thereby reconstructing the endocannabinoid-anti-inflammatory axis in a low-FAAH environment and ultimately suppressing liver fibrosis progression. Consequently, subsequent studies will focus on this key drug target to explore the underlying mechanisms, offering a novel direction for the development of drugs for hepatobiliary atrophy with cholangiolithiasis.

Based on WES, seven hub genes with high mutation rates were identified in patients with hepatobiliary atrophy associated with HL. Among these, TMEM150B, TNIP1, and FAAH exhibited notable commonality in mutations. The functions of FAAH, TMEM150B, TNIP1, ATRN, and FBXW4 were primarily linked to immune regulation. Notably, WNT8B may play a role in liver tissue atrophy and the transformation of malignant liver tissue. However, this study has several limitations. First, the use of peripheral blood rather than liver tissue for sequencing may have missed liver-specific genetic variants, and future validation using liver tissue sequencing is planned. Second, the small sample size may limit the detection of low-frequency mutations. Future research will expand the cohort and verify findings using public databases. Third, this study is primarily based on bioinformatic analyses and lacks experimental validation. Future work will include functional assays and tissue-level protein detection to explore the role of hub genes in Wnt and inflammatory pathways. Furthermore, the predicted regulatory networks and drug targets (such as Fostamatinib and FAAH) have not been experimentally confirmed; subsequent molecular and cellular studies will validate their regulatory mechanisms and therapeutic efficacy. Finally, the association between the identified genetic variants and long-term clinical outcomes has not been established, and future prospective cohort studies will further assess their clinical relevance.

## Conclusion

Our study identified seven hub genes (*TMEM150B*, *TNIP1*, *ATRN*, *FAAH*, *FBXW4*, *RAX*, and *WNT8B*) uniquely mutated in patients with hepatobiliary atrophy associated with HL. These genes are likely involved in the pathogenesis of the disease through their roles in inflammatory responses and the Wnt signaling pathway. The constructed lncRNA-miRNA-mRNA and TF-mRNA networks provided further insights into the regulatory mechanisms of these hub genes, emphasizing the important roles of specific lncRNAs, miRNAs, and TFs in modulating their expression. Additionally, fostamatinib was identified as a potential therapeutic agent targeting *FAAH*, demonstrating a favorable binding affinity of −9.2 kcal/mol, which offers a promising direction for future treatment strategies for HL. RT–qPCR analysis confirmed the differential expression of six of the seven hub genes—*TMEM150B*, *TNIP1*, *ATRN*, *FAAH*, *FBXW4*, and *WNT8B*—in HL samples, highlighting their potential importance in disease progression. Overall, this study provides novel insights into the molecular mechanisms underlying hepatobiliary atrophy with HL and identifies potential therapeutic targets for future research and clinical applications.

## Supplemental Information

10.7717/peerj.21059/supp-1 Supplemental Information 1 Supplementary Materials.

10.7717/peerj.21059/supp-2 Supplemental Information 2 MIQE checklist.

## Additional Information and Declarations

### Data Availability

The following information was supplied regarding data availability:

The data is available at the Genome Sequence Archive human repository: HRA009591.

Dan, Tang (2025). Groundbreaking Functional Network Analysis in Hepatobiliary Disorders: Identifying Novel Therapeutic Targets through Whole-Exome Sequencing. figshare. Journal contribution. https://doi.org/10.6084/m9.figshare.28817336.v3.
