# Supplementary material for: Functional network analysis in hepatolithiasis: identifying novel therapeutic targets through whole-exome sequencing
Source: PeerJ. 2026 Apr 9;14:e21059. doi: 10.7717/peerj.21059 (PMC13070319; doi:10.7717/peerj.21059)
Supplement: Supplemental Information 1 [file peerj-14-21059-s001.docx]

**Somatic Mutation Profiling in Liver Atrophy Patients with Hepatolithiasis through Whole-Exome Sequencing**

**Dan Tang^1,2,3^，XuanYu Gu^2^，Dan Liu^2^，JiaLi Yang^2^，Lijin Zhao^2*^**


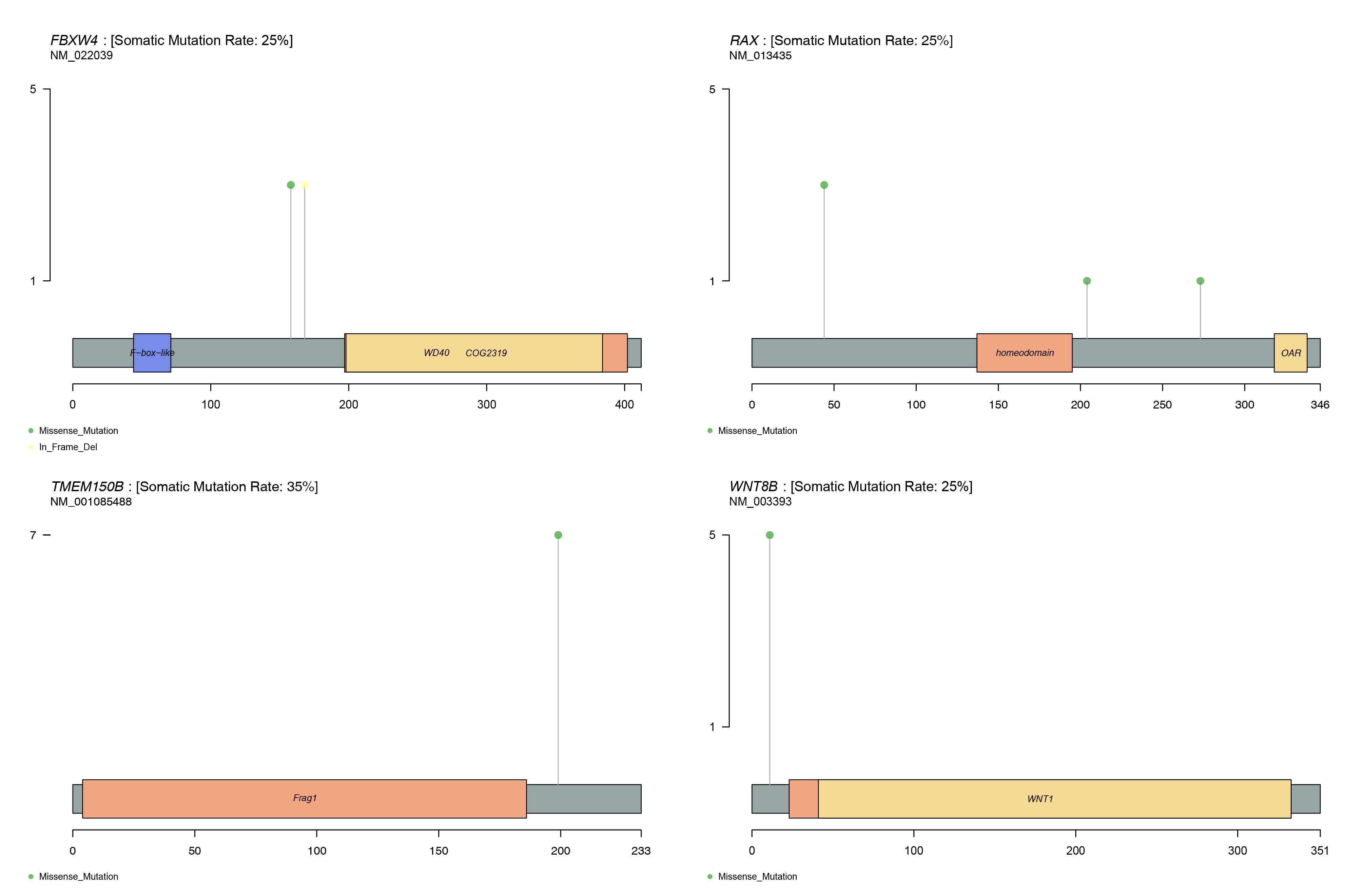


**Supplementary Fig. S1** A-D. Mutation sites within the other hub genes.

**Table S1: Reaction system for reverse transcription of mRNA.**

| **Component** | **Volume** |
| --- | --- |
| 5x Reaction Buffer | 4ul |
| Primer | 1ul |
| SweScript RT I Enzyme Mix | 1ul |
| Total RNA | 0.1ng-5ug |
| Nuclease-Free Water | Add to 20ul |

**Table S2: Reaction program for reverse transcription of mRNA.**

| **Temperature** | **Time** |
| --- | --- |
| 25℃ | 5min |
| 50℃ | 15min |
| 85℃ | 5s |
| 4℃ | hold |

**Table S3: Reaction program for qRT-PCR.**

|  | **Temperature** | **Time** |
| --- | --- | --- |
| Pre-denaturation | 95℃ | 1min |
| Denaturation | 95℃ | 20s |
| Annealing | 55℃ | 20s |
| Extension | 72℃ | 30s |

**Table S4: Information on qRT-PCR related instruments.**

| **Instruments** | **Manufacturers** |
| --- | --- |
| S1000™ Thermal Cycler General PCR Instrument | BIO-RAD |
| CFX Connect Real-Time Quantitative Fluorescence PCR Instrument | BIO-RAD |
| 96-well quantitative PCR plate | Servicebio |
| PCR plate sealing film | LABSELECT |
| Chemiluminescent Imaging System ChemiScope6100 | Shanghai Qinxiang Scientific Instrument Co. |
| H1 16KR Benchtop Freezer High Speed Centrifuge | Kasei Instruments |
| SC-3610 low speed centrifuge | Anhui Zhongke Zhongjia Scientific Instrument Co. |
| Handheld instantaneous centrifuge (for reagent preparation or mixing) | SCLLOGEX |
| Vortex Oscillator SCL-VS | SCLLOGEX |
| Electrophoresis apparatus (164-5050) | BIO-RAD |
| 10μl, 20μl, 200ul, 1000ul tips | Beijing Lanjieke Technology Co. |
| Micropipette | thermo scientific |
| Handheld Instant Centrifuge CF2800M | LABGIC |
| Grinder | Servicebio |
| Plate sealer | Servicebio |

**Table S5:** The result of FASTQ data analysis of the 20 specimens.

| Sample | Strand | Total Reads Count(#) | Total Bases Count(bp) | Average Read Length(bp) | Q20 Bases Count(bp) | Q20 Bases Ratio(%) | Q30 Bases Count(bp) | Q30 Bases Ratio(%) |
| --- | --- | --- | --- | --- | --- | --- | --- | --- |
| C1 | Read1 | 24531901 | 3111404443 | 127 | 3061600645 | 98.40% | 2962654020 | 95.22% |
| C1 | Read2 | 24531901 | 3112393983 | 127 | 3013173503 | 96.81% | 2848373501 | 91.52% |
| C2 | Read1 | 34360220 | 4328661518 | 126 | 4261717911 | 98.45% | 4127971120 | 95.36% |
| C2 | Read2 | 34360220 | 4329843326 | 126 | 4225043929 | 97.58% | 4028749168 | 93.05% |
| C3 | Read1 | 37822167 | 5033676185 | 133 | 4950951925 | 98.36% | 4788799962 | 95.14% |
| C3 | Read2 | 37822167 | 5034425812 | 133 | 4905223494 | 97.43% | 4670585329 | 92.77% |
| C4 | Read1 | 28385704 | 3467492882 | 122 | 3415364465 | 98.50% | 3308762489 | 95.42% |
| C4 | Read2 | 28385704 | 3468485498 | 122 | 3360652672 | 96.89% | 3177923312 | 91.62% |
| C5 | Read1 | 35734555 | 4550656153 | 127 | 4481124528 | 98.47% | 4342410652 | 95.42% |
| C5 | Read2 | 35734555 | 4551698790 | 127 | 4455026228 | 97.88% | 4265487986 | 93.71% |
| C6 | Read1 | 32161224 | 4212777557 | 131 | 4147344251 | 98.45% | 4014131459 | 95.28% |
| C6 | Read2 | 32161224 | 4213570459 | 131 | 4092049352 | 97.12% | 3880144939 | 92.09% |
| C7 | Read1 | 33482705 | 4201925659 | 125 | 4135724476 | 98.42% | 4003669741 | 95.28% |
| C7 | Read2 | 33482705 | 4203286478 | 126 | 4104860957 | 97.66% | 3919873040 | 93.26% |
| C8 | Read1 | 22925879 | 2840696097 | 124 | 2797047372 | 98.46% | 2707160178 | 95.30% |
| C8 | Read2 | 22925879 | 2841426801 | 124 | 2777821066 | 97.76% | 2653542789 | 93.39% |
| C9 | Read1 | 25689918 | 3291015530 | 128 | 3239392096 | 98.43% | 3134606472 | 95.25% |
| C9 | Read2 | 25689918 | 3291720011 | 128 | 3202227735 | 97.28% | 3042020578 | 92.41% |
| C10 | Read1 | 37871057 | 5218474665 | 138 | 5133445900 | 98.37% | 4962548933 | 95.10% |
| C10 | Read2 | 37871057 | 5219014511 | 138 | 5039515565 | 96.56% | 4743392201 | 90.89% |
| S1 | Read1 | 18274969 | 2712357965 | 148 | 2672881075 | 98.54% | 2590003073 | 95.49% |
| S1 | Read2 | 18274969 | 2712373728 | 148 | 2653609597 | 97.83% | 2534938788 | 93.46% |
| S2 | Read1 | 29928928 | 4034434365 | 135 | 3970457945 | 98.41% | 3841774402 | 95.22% |
| S2 | Read2 | 29928928 | 4034815783 | 135 | 3918368053 | 97.11% | 3712210872 | 92.00% |
| S3 | Read1 | 28700950 | 3592617056 | 125 | 3535977561 | 98.42% | 3423573280 | 95.29% |
| S3 | Read2 | 28700950 | 3593550341 | 125 | 3523313942 | 98.05% | 3383556551 | 94.16% |
| S4 | Read1 | 48268538 | 6523285836 | 135 | 6420766433 | 98.43% | 6217660009 | 95.31% |
| S4 | Read2 | 48268538 | 6523826236 | 135 | 6332502622 | 97.07% | 5993994669 | 91.88% |
| S5 | Read1 | 25510210 | 3687058275 | 145 | 3625510247 | 98.33% | 3503582900 | 95.02% |
| S5 | Read2 | 25510210 | 3687339719 | 145 | 3573380033 | 96.91% | 3382105160 | 91.72% |
| S6 | Read1 | 37390315 | 4763904155 | 127 | 4690373109 | 98.46% | 4539772574 | 95.30% |
| S6 | Read2 | 37390315 | 4764750001 | 127 | 4640268679 | 97.39% | 4409297778 | 92.54% |
| S7 | Read1 | 35691467 | 4650797594 | 130 | 4580979228 | 98.50% | 4440149197 | 95.47% |
| S7 | Read2 | 35691467 | 4651720792 | 130 | 4542796109 | 97.66% | 4335834115 | 93.21% |
| S8 | Read1 | 38534653 | 5125149905 | 133 | 5048208131 | 98.50% | 4887531666 | 95.36% |
| S8 | Read2 | 38534653 | 5125704425 | 133 | 4965380217 | 96.87% | 4686823506 | 91.44% |
| S9 | Read1 | 27714905 | 3528145652 | 127 | 3474692322 | 98.48% | 3364665192 | 95.37% |
| S9 | Read2 | 27714905 | 3528914930 | 127 | 3403830923 | 96.46% | 3197582673 | 90.61% |
| S10 | Read1 | 13511103 | 1843386605 | 136 | 1812105093 | 98.30% | 1748031526 | 94.83% |
| S10 | Read2 | 13511103 | 1843702411 | 136 | 1786856849 | 96.92% | 1686222011 | 91.46% |

**Table S6:** The table of sequencing sample alignment.

| Sample | Raw Reads (All reads) | Raw Data(Mb) | Mapped Reads | Fraction of Mapped Reads | Average depth | Coverage (>=4x) | Coverage (>=10x) | Coverage (>=30x) | Coverage (>=100x) |
| --- | --- | --- | --- | --- | --- | --- | --- | --- | --- |
| C1 | 49509358 | 6247.89 | 49030736 | 99.03% | 106.14 | 98.66% | 98.44% | 96.13% | 48.27% |
| C2 | 69569775 | 8704.4 | 68678424 | 98.72% | 147.96 | 98.69% | 98.52% | 97.15% | 71.73% |
| C3 | 76794813 | 10130.47 | 75589452 | 98.43% | 165.88 | 98.71% | 98.57% | 97.61% | 77.64% |
| C4 | 57286200 | 6963.78 | 56740751 | 99.05% | 121.07 | 98.68% | 98.50% | 96.95% | 59.68% |
| C5 | 73454576 | 9209.74 | 71426113 | 97.24% | 154.12 | 98.67% | 98.48% | 96.90% | 72.33% |
| C6 | 65519028 | 8491.06 | 64276656 | 98.10% | 137.99 | 98.88% | 98.70% | 97.00% | 66.13% |
| C7 | 67971485 | 8459.11 | 66923413 | 98.46% | 143.34 | 98.86% | 98.69% | 97.32% | 69.89% |
| C8 | 46214186 | 5701.57 | 45834266 | 99.18% | 101.13 | 98.68% | 97.62% | 86.94% | 40.71% |
| C9 | 51690068 | 6599.25 | 51348169 | 99.34% | 110.4 | 98.68% | 98.51% | 96.69% | 51.49% |
| C10 | 77206316 | 10517.01 | 75673480 | 98.01% | 163.83 | 98.72% | 98.55% | 96.96% | 72.43% |
| S1 | 36617724 | 5428.09 | 36532003 | 99.77% | 89.98 | 98.56% | 98.14% | 92.00% | 34.97% |
| S2 | 60160884 | 8085.53 | 59821282 | 99.44% | 137.34 | 98.66% | 98.44% | 96.35% | 65.01% |
| S3 | 57726882 | 7203.47 | 57373631 | 99.39% | 129.25 | 98.63% | 98.40% | 96.38% | 63.01% |
| S4 | 97452097 | 13096.8 | 96460871 | 98.98% | 219.63 | 98.67% | 98.40% | 95.93% | 75.94% |
| S5 | 51621587 | 7404.69 | 50965176 | 98.73% | 117.88 | 98.58% | 98.29% | 95.37% | 53.49% |
| S6 | 75126268 | 9546.98 | 74742598 | 99.49% | 164.84 | 98.70% | 98.56% | 97.64% | 74.61% |
| S7 | 71808724 | 9325.16 | 71335290 | 99.34% | 160.66 | 98.67% | 98.51% | 97.22% | 75.20% |
| S8 | 77435025 | 10270.33 | 77011851 | 99.45% | 168.27 | 98.72% | 98.54% | 96.86% | 73.00% |
| S9 | 55703424 | 7071.64 | 55397168 | 99.45% | 121.16 | 98.82% | 98.45% | 93.88% | 52.34% |
| S10 | 27356914 | 3705 | 27002728 | 98.71% | 58.18 | 98.43% | 96.86% | 76.98% | 12.59% |

**Table S7:** Enrichment results of core gene pathways.

| Category | GO | Description | PARENT_GO | Log10（P） | Enrichment | Z-score | TotalGeneInLibrary | GeneInGO | GeneInHitList | GeneInGOAndHitList | GeneID | Hits |
| --- | --- | --- | --- | --- | --- | --- | --- | --- | --- | --- | --- | --- |
| GO Biological Processes | GO:0016055 | Wnt signaling pathway | 19_GO:0023052 signaling | -2.8 | 31 | 7.6 | 30182 | 282 | 7 | 2 | 6468\|7479 | FBXW4\|WNT8B |
| GO Biological Processes | GO:0198738 | cell-cell signaling by wnt | 19_GO:0023052 signaling | -2.8 | 31 | 7.6 | 30182 | 282 | 7 | 2 | 6468\|7479 | FBXW4\|WNT8B |
| GO Biological Processes | GO:1905114 | cell surface receptor signaling pathway involved in cell-cell signaling | 19_GO:0023052 signaling | -2.5 | 23 | 6.5 | 30182 | 377 | 7 | 2 | 6468\|7479 | FBXW4\|WNT8B |
| GO Biological Processes | GO:0071396 | cellular response to lipid | 19_GO:0050896 response to stimulus | -2.2 | 16 | 5.4 | 30182 | 527 | 7 | 2 | 7479\|10318 | WNT8B\|TNIP1 |
| GO Biological Processes | GO:0006954 | inflammatory response | 19_GO:0050896 response to stimulus | -2.2 | 16 | 5.3 | 30182 | 541 | 7 | 2 | 8455\|10318 | ATRN\|TNIP1 |
| KEGG Pathway | hsa04550 | Signaling pathways regulating pluripotency of stem cells |  | -1.5 | 30 | 5.3 | 30182 | 143 | 7 | 1 | 7479 | WNT8B |
| KEGG Pathway | hsa05226 | Gastric cancer |  | -1.5 | 29 | 5.2 | 30182 | 149 | 7 | 1 | 7479 | WNT8B |
| KEGG Pathway | hsa05225 | Hepatocellular carcinoma |  | -1.4 | 26 | 4.9 | 30182 | 168 | 7 | 1 | 7479 | WNT8B |
| KEGG Pathway | hsa04310 | Wnt signaling pathway |  | -1.4 | 25 | 4.9 | 30182 | 170 | 7 | 1 | 7479 | WNT8B |
| KEGG Pathway | hsa05205 | Proteoglycans in cancer |  | -1.3 | 21 | 4.4 | 30182 | 205 | 7 | 1 | 7479 | WNT8B |
